# Immunomodulatory Hydrogel Coating with SeNPs and Lithium Silicate Synergistically Promotes Osseointegration and Prevents Infection on Titanium Implants

**DOI:** 10.1002/advs.202513195

**Published:** 2025-12-12

**Authors:** Su Jiang, Baisheng Cai, Cong Ye, Kefan Wu, Kuan Liu, Pengcheng Xu, Fan Liu, Yake Liu

**Affiliations:** ^1^ Department of Orthopaedics Affiliated Hospital of Nantong University Medical School of Nantong University Nantong 226001 China; ^2^ Binhai County People's Hospital Yancheng Jiangsu 224500 China; ^3^ Yancheng Dafeng People's Hospital Yancheng Jiangsu 224100 China; ^4^ Department of Orthopaedics The First Affiliated Hospital of Huzhou University Huzhou 313000 China

**Keywords:** anti‐infective properties, bone regeneration, implant coating, lithium magnesium silicate, SeNPs

## Abstract

Effective osseointegration of implants remains a major challenge in biomaterials and regenerative medicine. In this context, restoring the disrupted microenvironment between the implant and bone tissue is critical, as it is primarily influenced by excessive ROS, infections, immune inflammatory reactions, and imbalances in bone homeostasis. To address these challenges, a composite hydrogel coating composed of lithium disilicate (Lap) and selenium nanoparticles (SeNPs) is developed, fabricated through the crosslinking of methacrylated carboxymethyl chitosan (CMCSMA) and methacrylated gelatin hydrogel (GelMA). The resulting Lap‐CMCSMA/GelMA@SeNPs hydrogel exhibits excellent biocompatibility and forms a robust adhesion to titanium (Ti) implants. In vitro studies demonstrate that titanium substrates coated with Lap‐CMCSMA/GelMA@SeNPs could efficiently neutralize excessive intracellular ROS. Moreover, the coating displays potent anti‐inflammatory properties by promoting a shift in macrophage polarization toward the M2 phenotype. Additionally, the integration of SeNPs markedly improves the antibacterial performance of Lap, showing strong inhibitory effects against common pathogens such as *E. coli* and *S. aureus*. Both in vitro and in vivo evaluations show superior osteogenic activity of the Lap‐CMCSMA/GelMA@SeNPs‐coated implants, largely attributed to the inherent osteogenic potential of Lap. The findings indicate that titanium implants functionalized with the Lap‐CMCSMA/GelMA@SeNPs hydrogel may provide an innovative therapeutic strategy for mitigating peri‐implant microenvironment imbalances post‐surgery.

## Graphical abstract

This study introduces a multifunctional hydrogel coating (Lap‐CMCSMA/GelMA@SeNPs) that scavenges ROS, modulates immune responses, and shows strong antibacterial activity. It effectively restores the peri‐implant microenvironment. The coating exhibits excellent biocompatibility and promotes osteogenic differentiation. It also enhances in vivo bone integration. This approach represents a promising strategy to improve titanium implant performance.

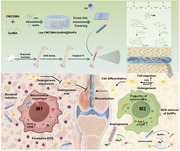

## 1 Introduction

Titanium, an inert metal, demonstrates superior biocompatibility and mechanical robustness, and corrosion resistance. As a result, titanium and its alloys have become critical materials for implants in orthopedics and dentistry.^[^ ^1^ ^]^ However, many factors can affect the perfect integration of titanium implants with bone tissue.^[^ ^2^, ^3^ ^]^ After titanium alloy internal fixation or prosthesis implantation, excessive accumulation of reactive ROS occurs in the local microenvironment, and oxidative stress (OS) can cause damage to bone cells and bone tissue, which is not conducive to maintaining the stability of the microenvironment.^[^ ^4^ ^]^ In addition, titanium implants themselves do not have antibacterial properties. Improper surgical operations often cause postoperative infections, which can form dense biofilms on the implants, which is not conducive to osseointegration,^[^ ^5^ ^]^ this not only prolongs the hospital stay of patients but also increases their economic burden. Due to the body's innate immune rejection, acute or severe inflammatory reactions often occur around the internal fixation or prosthesis, further hindering the osteogenesis process in the medullary cavity, ultimately leading to poor osseointegration.^[^ ^6^, ^7^, ^8^ ^]^ Therefore, how to prepare coatings that have sufficient adhesion and osteoinductive activity, angiogenic effects, as well as anti‐inflammatory, antioxidative, and antimicrobial capabilities within the osteogenic microenvironment has become a key direction to break through the existing bottlenecks.

Currently, common implant modifications encompass physical, chemical, biomaterial‐based, or multifunctional composite approaches.^[^ ^3^, ^9^ ^]^ Sun et al.^[^ ^10^ ^]^ developed a gelatin‐alginate hydrogel crosslinked by TGase and loaded with antibiotics that effectively delivered antibiotics, thereby reducing bacterial attachment and biofilm formation at the implant surface. Che et al.^[^ ^11^ ^]^ utilized 3D printing to construct titanium alloy scaffolds coated with a hydrogel incorporating vascular endothelial growth factor (VEGF) and bone morphogenetic protein‐9 (BMP‐9). Lee et al.^[^ ^12^ ^]^ constructed a β‐TCP/hydrogel‐coated titanium implant loaded with BMP‐2, which exhibited superior osteogenic performance in the rabbit tibial medullary cavity. While these aforementioned surface coating modification strategies specifically addressed issues such as bacterial infection or promoted osteogenic mineralization and enhanced osseointegration, they failed to comprehensively account for the complex and imbalanced microenvironment encountered during bone repair processes.

In recent years, multifunctional hydrogels have garnered significant attention in the field of biomaterials due to their biomimetic microenvironment, controllable drug release, and potential for bioactive modification.^[^ ^13^, ^14^, ^15^, ^16^, ^17^ ^]^ The use of hydrogel coatings has also become increasingly prevalent. Notably, methacrylated carboxymethyl chitosan (CMCSMA) and methacrylated gelatin hydrogel (GelMA) have demonstrated exceptional performance in biomedical applications. CMCSMA is synthesized by modifying carboxymethyl chitosan (CMCS) with methacrylic anhydride. CMCS itself is derived from chitosan (CS) through the introduction of carboxymethyl (CM) groups,^[^ ^18^, ^19^ ^]^ CMCSMA preserves the favorable characteristics of chitosan (CS), including biocompatibility, biodegradability, and natural antibacterial properties, but also introduces photocurable capability, enhanced mechanical strength, and sustained drug release characteristics.^[^ ^20^ ^]^ GelMA, prepared by chemically modifying gelatin with methacrylic anhydride, retains essential bioactive domains such as the RGD motif and cleavage sites for matrix metalloproteinases (MMPs). Moreover, it demonstrates superior biocompatibility and significantly enhances cell attachment, growth, and differentiation.^[^ ^21^, ^22^, ^23^, ^24^ ^]^

Selenium (Se) is an essential trace element that plays a crucial role in human physiological processes and is involved in various metabolic activities.^[^ ^25^ ^]^ However, concerns regarding the toxicological safety of organic and inorganic selenium compounds limit their clinical applications. In comparison, SeNPs exhibit lower biological toxicity and higher biocompatibility than their organic and inorganic counterparts. SeNPs also possess the ability to modulate biological immune activity, including antibacterial, antiviral, and anticancer properties, as well as significant antioxidant and anti‐inflammatory effects.^[^ ^26^, ^27^, ^28^, ^29^, ^30^ ^]^ Beyond their antioxidant capacity, SeNPs exert multiple biological functions that help modulate oxidative homeostasis, attenuate bone degradation, and accelerate regenerative bone formation.^[^ ^31^ ^]^ Lap (Li_2_Mg_2_O_9_Si_3_), a layered silicate structurally similar to lithium soapstone, the as‐synthesized nanocrystals display a disk‐like configuration, featuring an average lateral dimension of ≈25 nm and a thickness of ≈0.9 nm, consistent with their layered crystal structure. Despite its insolubility in both water and organic solvents, it has the ability to self‐assemble into colloidal suspensions or form gel‐phase aggregates in aqueous environments.^[^ ^32^ ^]^ The release of silicate ions and Mg^2^⁺ from Lap has been demonstrated to enhance vascular regrowth.^[^ ^33^ ^]^ Lap has been shown to promote osteogenic differentiation of human mesenchymal stem cells (hMSCs) in the absence of external osteoinductive agents,^[^ ^34^ ^]^ promote osteogenic induction of bone marrow stromal cell (BMSC) differentiation via activation of the PI3K‐Akt signaling pathway.^[^ ^35^, ^36^ ^]^Additionally, Lap can adsorb inflammatory mediators such as cytokines, thereby synergistically reducing local inflammation when combined with SeNPs. The release of Mg^2+^ further contributes to its antibacterial effects.^[^ ^37^, ^38^ ^]^

**Figure 1:**
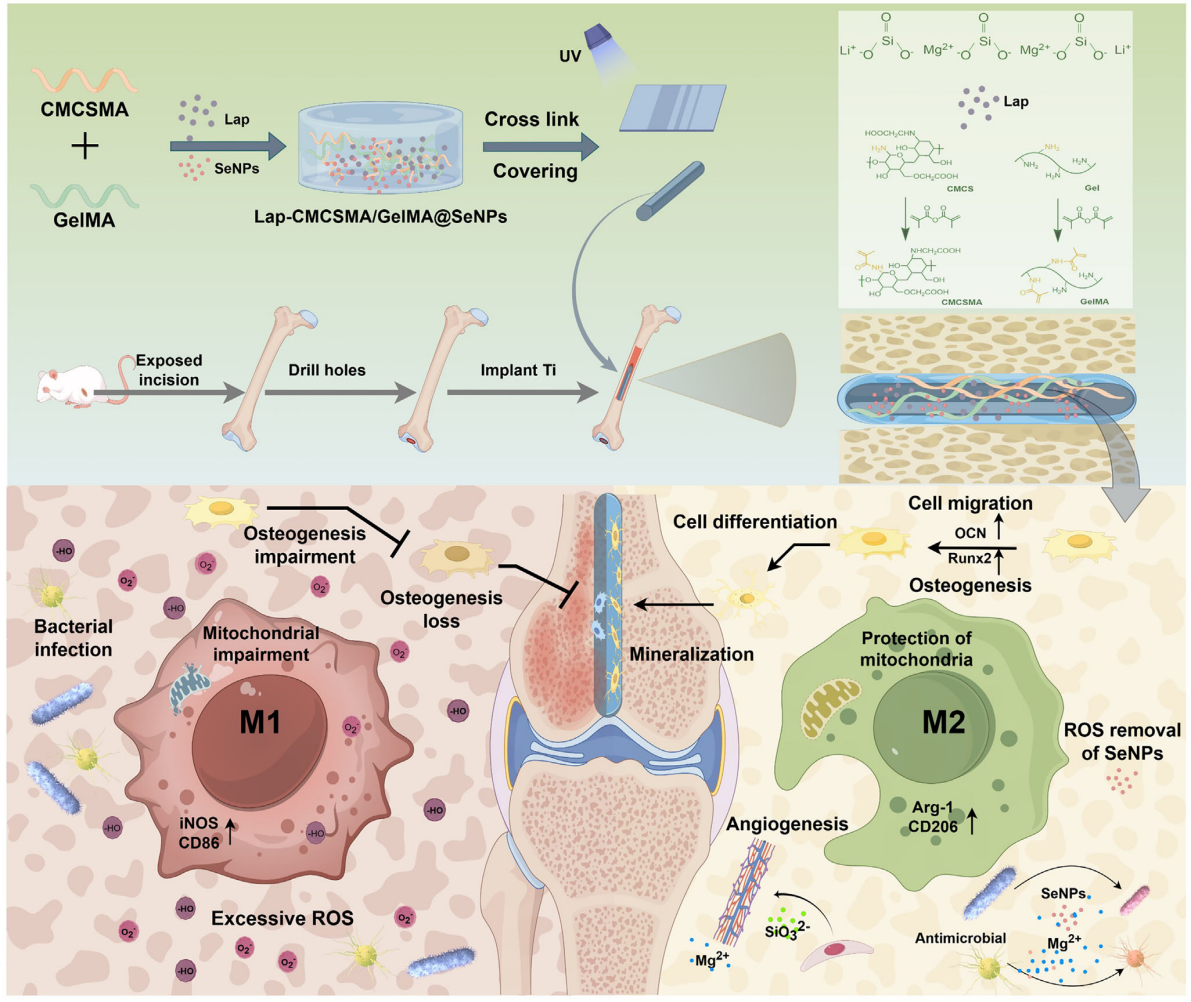
This figure illustrates the fabrication process of the Lap‐CMCSMA/GelMA@SeNPs composite hydrogel coating, which demonstrates antibacterial activity, ROS scavenging capability, anti‐inflammatory properties, angiogenic potential, and osteogenic functionality.

In this study, a Lap‐CMCSMA/GelMA@SeNPs hydrogel coating was successfully constructed. In contrast to conventional “dual‐functional” coatings that address only one or two biological requirements, the current design simultaneously targets five key therapeutic functions: immunomodulation‐centered ROS scavenging, angiogenesis promotion, bone regeneration, and antibacterial activity, thereby enabling a multi‐faceted and coordinated therapeutic intervention within the complex pathological microenvironment. The methacryloyl groups in CMCSMA and GelMA can form a cross‐linked network via free radical polymerization under the action of a photoinitiator, thereby endowing the hydrogel with enhanced structural stability. The remaining functional groups in carboxymethyl chitosan and gelatin are capable of forming hydrogen bonds or electrostatic attractions with the hydroxyl groups (TiO_2_‐OH) present onto the titanium surface, thereby ensuring robust adhesion.^[^ ^39^, ^40^ ^]^ This coating establishes a pro‐osteogenic microenvironment on the titanium implant surface, simultaneously exhibiting antibacterial properties, immunomodulatory functions, and osteogenic induction capabilities. In vitro studies were conducted to assess the coating's antibacterial activity against *E. coli* and *S. aureus*. Its biocompatibility was examined via co‐culture with MC3T3 cells and HUVECs. The osteogenic capability was demonstrated through experiments involving bone marrow mesenchymal stem cells (BMSCs), in contrast, experiments involving macrophage cultures confirmed that the coating can induce macrophage polarization toward the M2 phenotype. In vivo, a rat model of femoral distal bone defect with intramedullary nail implantation was employed to confirm the coating's capacity to suppress bone tissue inflammation and promote bone repair (**Figure** **1**).

## 2 Result

### 2.1 Synthesis of Hydrogel Coatings

**Figure 2:**
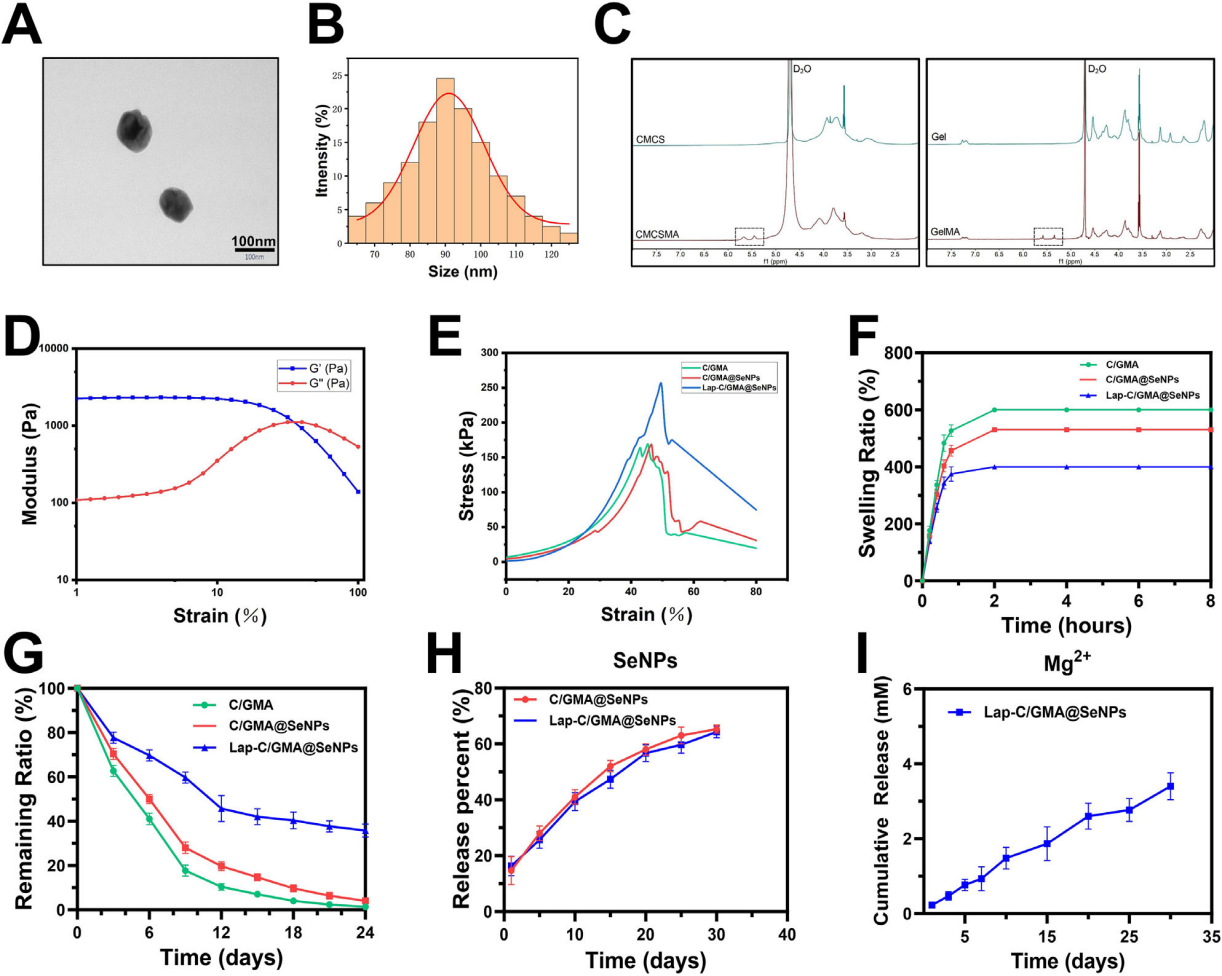
Preparation and characterization of hydrogel coatings. A) TEM image of the SeNPs stock solution. B) Particle size distribution histogram of SeNPs. C) ^1^H NMR spectra of CMCS, CMCSMA, Gel, and GelMA. D) Rheological behavior of the Lap‐C/GMA@SeNPs hydrogel. E) Typical stress–strain behavior of hydrogels with varying formulations. F) Swelling ratios of each hydrogel component in phosphate‐buffered saline (PBS). G) Degradation profiles of each hydrogel component in PBS. H) Analysis of selenium nanoparticle release dynamics from hydrogels. I) Release kinetics of Mg^2+^ from hydrogels. (*n* = 3; data are expressed as mean ± SD. ^*^ *p* < 0.05, ^**^ *p* < 0.01, ^***^ *p* < 0.001, ns indicates no significant difference).

The composite hydrogel coating (Lap‐CMCSMA/GelMA@SeNPs) is abbreviated as (Lap‐C/GMA@SeNPs) throughout this article and in the Figures. The SeNPs incorporated into the coating exhibit excellent dispersion in solution and are predominantly spherical with diameters ranging from 80 to 100 nm, as confirmed by TEM (**Figure** **2**A,B). As illustrated in Figure 2C, 1H NMR analysis revealed new characteristic peaks for CMCSMA and GelMA groups between 5.3 and 6.0 ppm, resulting from the vinyl protons associated with the methacrylate moiety in methacrylic anhydride (MA).

The rheological properties of the C/GMA, C/GMA@SeNPs, and Lap‐C/GMA@SeNPs hydrogels were investigated to elucidate the effects of SeNPs and Laponite incorporation on their viscoelastic behavior (Figure S1, Supporting Information). All samples exhibited typical viscoelastic characteristics, where the elastic modulus (G′) remained higher than the viscous modulus (G″) throughout the linear viscoelastic range, suggesting a primarily elastic behavior and the establishment of a robust, crosslinked structure. The C/GMA hydrogel displayed a moderate G′ value and a G′–G″ crossover at approximately 50–60% strain, suggesting partial network breakdown under high deformation. Upon incorporation of SeNPs, both G′ and the critical strain increased, demonstrating that the nanoparticles functioned as additional physical crosslinking points, thereby enhancing the structural integrity of the network. Notably, the Lap‐C/GMA@SeNPs hydrogel exhibited the highest G′ and the broadest LVR, maintaining an elastic‐dominant response up to large strain levels (>80%), which can be attributed to the synergistic reinforcement provided by Laponite nanosheets and SeNPs. These findings are consistent with the compressive stress–strain behavior, confirming that the dual incorporation of SeNPs and Laponite effectively enhances both small‐strain stiffness and large‐strain mechanical resilience of the hydrogel coatings (Figure 2D).

All hydrogels exhibited typical elastic–plastic deformation profiles, characterized by an initial linear elastic region accompanied by a steady rise in stress as strain increases. The pristine C/GMA hydrogel showed the lowest compressive strength and fractured at a relatively low strain, reflecting limited network rigidity. With the addition of SeNPs, both compressive stress and fracture strain significantly increased, indicating that SeNPs acted as multifunctional nodes for physical crosslinking and energy dissipation, thereby improving the toughness and structural stability of the hydrogel. Remarkably, the Lap‐C/GMA@SeNPs hydrogel demonstrated the highest compressive modulus and peak stress among all groups, retaining structural integrity even under large deformations. This significant enhancement results from the synergistic interplay between Lap and SeNPs, which collectively enhanced crosslinking density and improved load transfer efficiency within the polymer matrix. These results align well with the rheological data, further confirming that the combined integration of SeNPs and Lap significantly reinforces the mechanical robustness and deformation tolerance of the hydrogel coatings (Figure 2E).

Swelling experiments demonstrated that after 0.8 h, the C/GMA hydrogel swelled to 5.26 times its original volume, the C/GMA@SeNPs hydrogel swelled to 4.65 times, and the Lap‐C/GMA@SeNPs hydrogel swelled to 3.70 times. These findings suggest that adding SeNPs decreases the hydrogel's swelling rate, and the addition of Lap further decreases the swelling rate. Hydrogels with lower swelling rates possess a tighter polymer network and higher mechanical strength, providing a more stable matrix for the adsorption and fixation of cell adhesion proteins including fibronectin and laminin, thus enhancing cell attachment (Figure 2F). We found that the residual mass percentages of the C/GMA, C/GMA@SeNPs, and Lap‐C/GMA@SeNPs hydrogels on the Ti surface were 62.50 ± 2.42%, 70.50 ± 2.60%, and 77.50 ± 2.50% after 3 days, respectively. The degradation rate significantly accelerated over subsequent days, with residual mass percentages of 10.50 ± 1.50%, 20.0 ± 2.0%, and 44.50 ± 5.50% after 12 days, respectively. Notably, after 24 days, the Lap‐C/GMA@SeNPs hydrogel retained 35.70 ± 2.95% of its initial mass, suggesting its potential for controlled drug release (Figure 2G). Inductively coupled plasma (ICP) analysis was employed to measure selenium (Se) release from C/GMA@SeNPs and Lap‐C/GMA@SeNPs hydrogels. The data revealed that the Se release rate from Lap‐C/GMA@SeNPs was marginally reduced compared to C/GMA@SeNPs, due to the delaying effect of Lap. Furthermore, the release of Mg^2+^ from the Lap‐C/GMA@SeNPs hydrogel was gentle and sustained over 30 days, enabling long‐term sustained release in vitro (Figure 2H,I).

**Figure 3:**
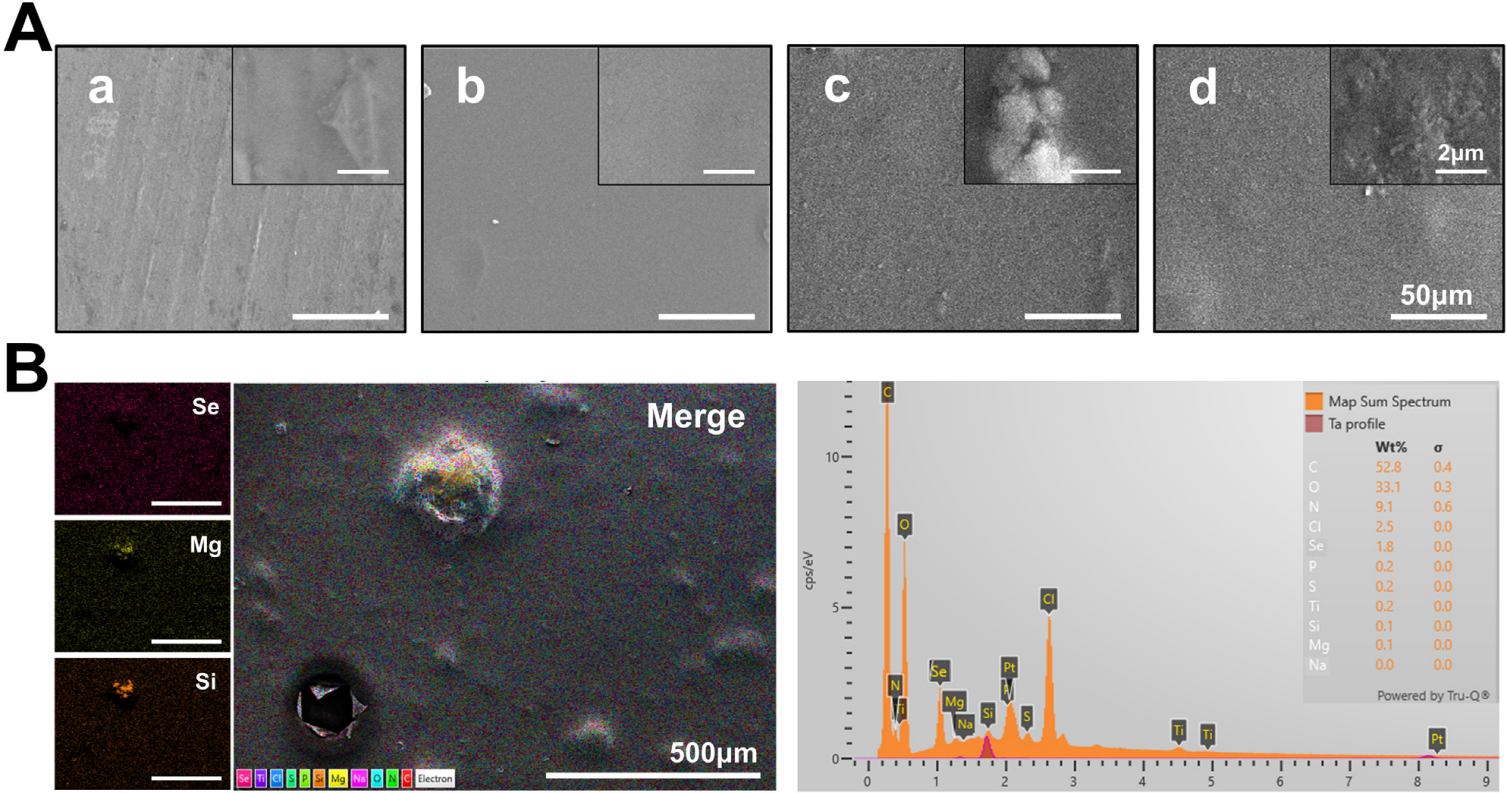
Characterization of hydrogel coatings. A) SEM images of titanium plates coated with different hydrogel components. B) Energy‐dispersive X‐ray spectroscopy (EDS) analysis of Lap‐C/GMA@SeNPs. (*n* = 3; data are expressed as mean ± SD. ^*^ *p* < 0.05, ^**^ *p* < 0.01, ^***^ *p* < 0.001, ns indicates no significant difference).

A comparison of the surface morphologies of various samples revealed that the polished pure Ti surface was relatively rough, whereas the Ti plate coated with the hydrogel (C/GMA) exhibited a uniform and dense coating. Upon the addition of SeNPs and Lap, the coating became rougher yet remained dense (**Figure** **3**A). EDS characterization identified Se, Mg, Si, and Li elements distributed within the Ti‐Lap‐C/GMA@SeNPs composite layer, validating the incorporation of both Lap and SeNPs, which were evenly distributed across the titanium substrate (Figure 3B).

### 2.2 Assessment of the in vitro Biocompatibility of Hydrogel‐Based Coatings

**Figure 4:**
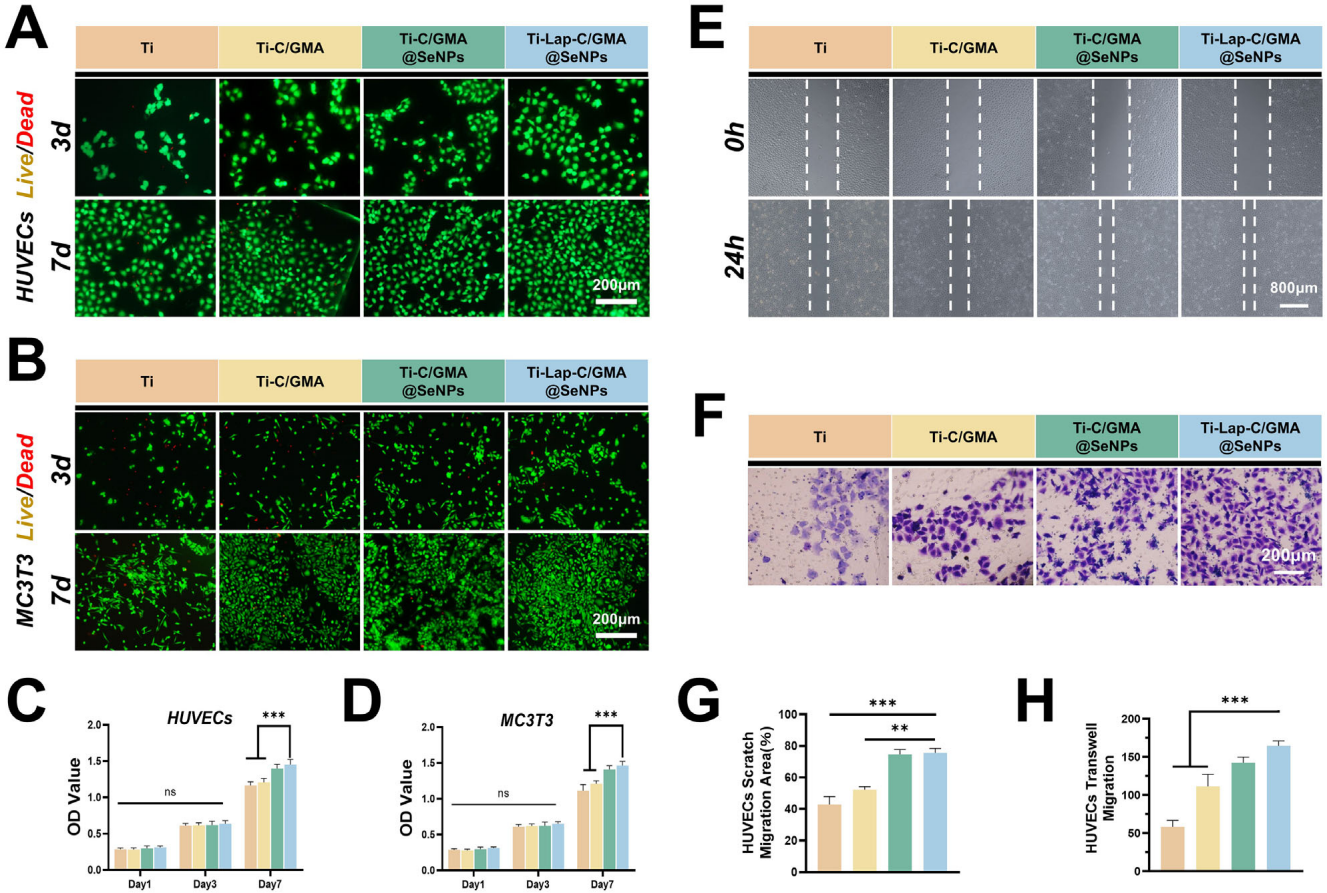
Biocompatibility assessment of hydrogel coatings. A) Live/dead staining images of HUVECs co‐cultured with hydrogel coatings on days 3 and 7. B) Live/dead staining images of MC3T3 cells co‐cultured with hydrogel coatings on days 3 and 7. C,D) CCK‐8 analysis of cell viability for cultures co‐cultured with different hydrogel coating components on days 1, 3, and 7. E) Wound healing migration assay of HUVECs.F) Transwell invasion assay of HUVECs. G,H) Comparative quantification of cellular migration and invasion in scratch wound and Transwell chamber assays. (*n =* 3; data are expressed as mean ± SD. ^*^ *p* < 0.05, ^**^ *p* < 0.01, ^***^ *p* < 0.001, ns indicates no significant difference).

High‐dose SeNPs can produce reactive oxygen species (including ‐OH, H_2_O_2_, O_2_^−^) via the Fenton reaction, leading to oxidative stress. To determine the optimal drug concentration, HUVECs and MC3T3 cells were co‐cultured with hydrogels containing different concentrations of SeNPs. CCK‐8 assay results demonstrated that a SeNPs concentration of 30 µg mL^−1^ promoted cell proliferation. Similarly, excessive Lap may lead to high Mg^2^⁺ concentrations, which could competitively inhibit calcium channels (e.g., NMDA receptors) and interfere with cell signaling. HUVECs and MC3T3 cells were also co‐cultured with hydrogels containing various Lap concentrations. CCK‐8 data revealed that a Lap concentration of 5 mg mL^−1^ enhanced cell proliferation. Based on these findings, a hydrogel coating was prepared using 30 µg mL^−1^ SeNPs and 5 mg mL^−1^ Lap (Figure S3, Supporting Information). Representative fluorescence micrographs showing live/dead cell staining results further confirmed that this hydrogel coating effectively promoted cell adhesion and exhibited excellent biocompatibility. For both HUVECs and MC3T3 cells, the majority of cells displayed green fluorescence, reflecting their viability, whereas a minimal number exhibited red fluorescence, indicative of cell death. Notably, the Ti‐Lap‐C/GMA@SeNPs group exhibited significantly higher cell numbers compared to other groups (**Figure** **4**A,B). Following a 7‐day culture period, the Ti‐Lap‐C/GMA@SeNPs group exhibited a markedly higher cell viability relative to the other groups, indicating that hydrogel coating enhanced the proliferation of HUVECs and MC3T3 cells via sustained release of SeNPs and Lap. (Figure 4C,D). To further validate these findings, scratch assays demonstrated that the Ti‐Lap‐C/GMA@SeNPs intervention group exhibited the highest cell migration rate within 24 h, while Transwell assays showed that this group had the largest number of cells passing through the membrane within 12 h (Figure 4E–H). In conclusion, Ti‐Lap‐C/GMA@SeNPs exhibits excellent biocompatibility and effectively regulates the release of SeNPs and Lap, thereby influencing cell behavior.

### 2.3 Evaluation of the in vitro Antibacterial Performance of Hydrogel Coating

*S. aureus* and *E. coli* are among the most prevalent pathogens in clinical settings. Typical confocal fluorescence images displaying live/dead staining of *E. coli* after 18 h of culture on various hydrogel surfaces. Green fluorescence (SYTO9) indicates viable bacteria, whereas red fluorescence (PI) represents dead bacteria.

**Figure 5:**
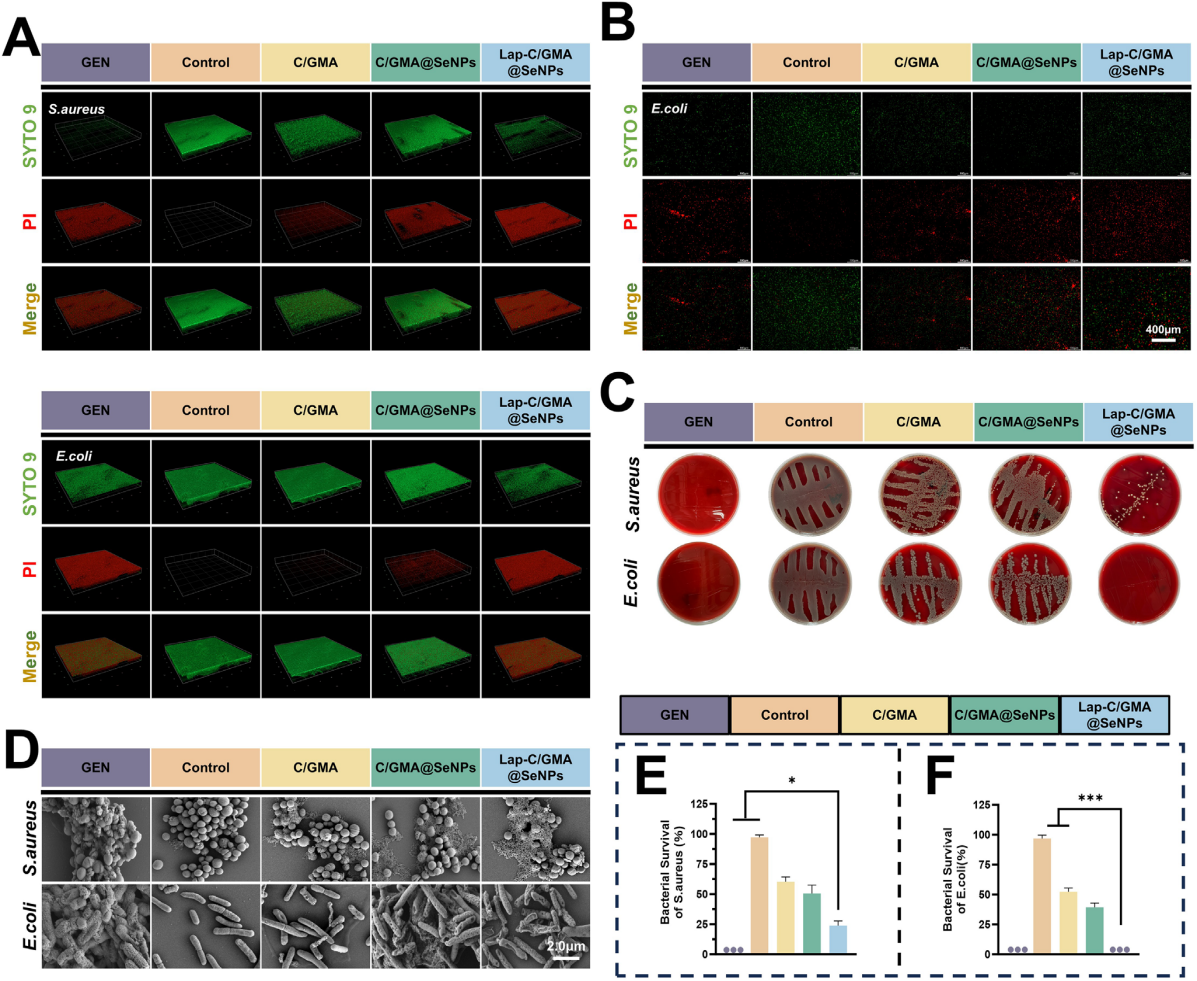
Assessment of the antibacterial performance of hydrogel coatings, encompassing the control group and the group treated with gentamicin. A) Live/dead staining of bacterial biofilms following co‐cultivation with the hydrogels, including the gentamicin‐treated positive control and untreated control groups. B) Viability staining of *E. coli* on hydrogel surfaces, including the gentamicin‐treated positive control and untreated groups. C) Antimicrobial activity of hydrogels with various components was evaluated using blood agar plate assays, exhibiting antibacterial performance toward S. aureus and E. coli through co‐culture experiments. D) Representative SEM micrographs of S. aureus and E. coli after exposure to hydrogels with varying formulations. E,F) Morphological observation of S. aureus and E. coli by scanning electron microscopy after treatment with various hydrogel systems. (*n =* 3; data are expressed as mean ± SD. ^*^ *p* < 0.05, ^**^ *p* < 0.01, ^***^ *p* < 0.001, ns indicates no significant difference).

3D reconstructions from confocal laser scanning microscopy (CLSM) depict biofilms formed by *S. aureus* and *E. coli* after co‐cultivation with various hydrogels. Both control and C/GMA groups exhibited well‐developed biofilms, showing a dominant green fluorescence pattern, whereas significantly disrupted and thinner biofilms with extensive red fluorescence were detected in the C/GMA@SeNPs and Lap‐C/GMA@SeNPs groups. The Lap‐C/GMA@SeNPs hydrogel demonstrated the most pronounced inhibitory effect against biofilm formation for both bacterial strains (**Figure** **5**A).

The control and C/GMA groups exhibited abundant green fluorescence, indicating substantial bacterial viability, while the C/GMA@SeNPs group displayed an increased proportion of red fluorescence. Notably, the Lap‐C/GMA@SeNPs group showed predominantly red fluorescence comparable to that of the gentamicin‐treated group, indicating potent bactericidal activity; a comparable pattern was likewise evident in the live/dead staining results of *S. aureus* (Figure 5B; Figure S4, Supporting Information).

To evaluate the in vitro antibacterial activity of Lap‐C/GMA@SeNPs against these pathogens, a blood agar plate counting method was employed. The findings revealed that Lap‐C/GMA@SeNPs showed strong antibacterial efficacy against both *E. coli* and *S. aureus*. Compared to the C/GMA@SeNPs intervention group, Lap‐C/GMA@SeNPs showed significantly enhanced antibacterial efficacy, the observed effect is presumed to originate from a synergistic mechanism involving the concurrent release of SeNPs and Mg^2^⁺ ions (Figure 5C). Bacterial morphology after co‐culture was further examined using SEM. Bacterial quantification revealed that the Lap‐C/GMA@SeNPs group exhibited a significantly lower level of viable microorganisms than its C/GMA@SeNPs counterpart, highlighting the superior antibacterial capacity of the Lap‐modified coating. (Figure 5D). Additionally, SEM images revealed more dead bacteria with surface dents and membrane collapse in the Lap‐C/GMA@SeNPs group. Quantitative assessment further confirmed the strong antibacterial activity of Lap‐C/GMA@SeNPs (Figure 5E,F).

### 2.4 In Vitro Investigation into the Antioxidative and Anti‐Inflammatory Performance of Hydrogel‐Based Coatings

**Figure 6:**
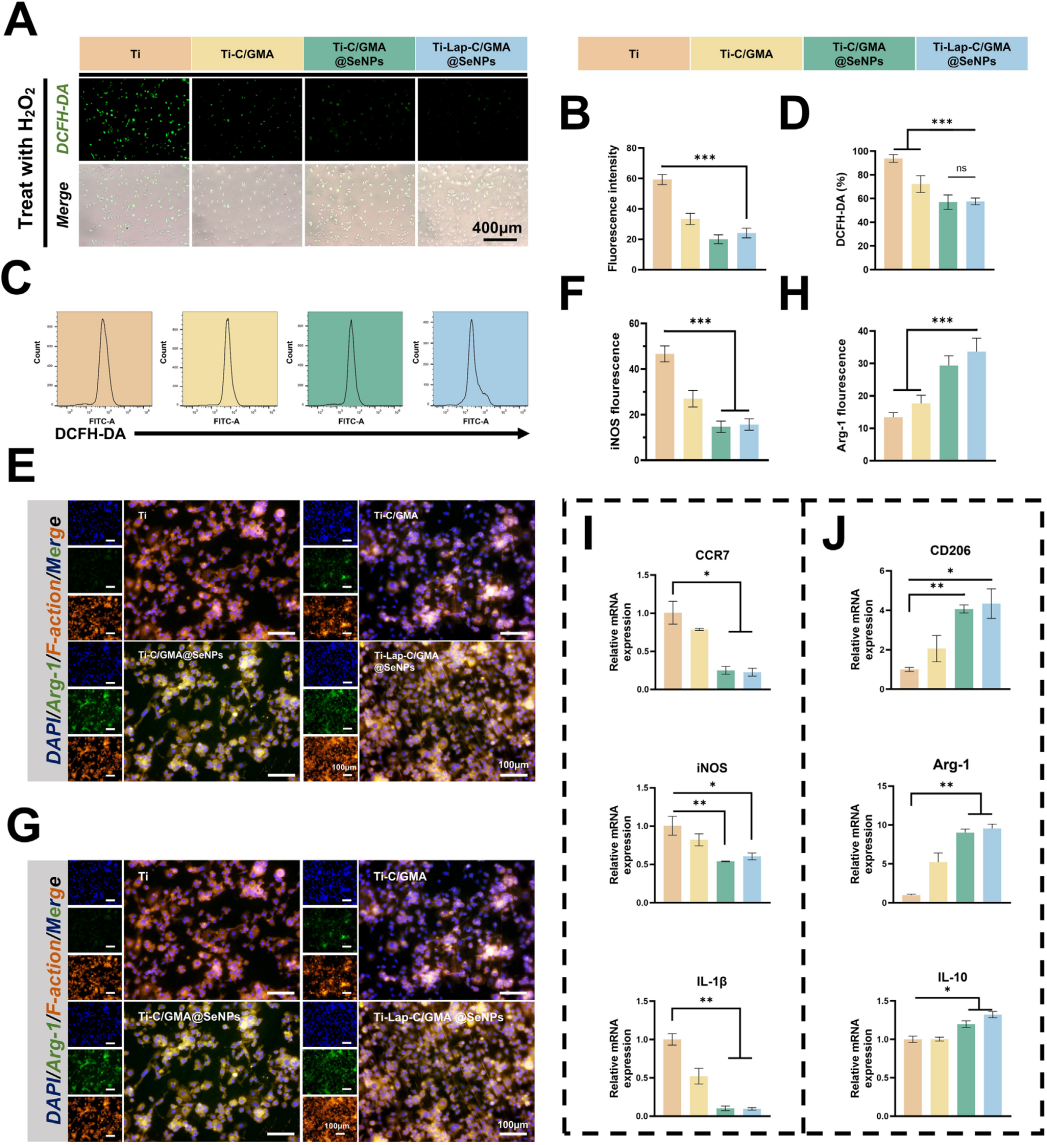
Assessment of the in vitro antioxidant and anti‐Inflammatory properties of hydrogel coatings. A) ROS fluorescence patterns observed in MC3T3 cells co‐cultured with titanium coatings bearing diverse hydrogel formulations. B) Quantification of ROS fluorescence intensity from staining results. C) Flow cytometry images of MC3T3 cells co‐cultured with titanium sheets coated with different hydrogel components. D) Quantitative flow cytometry analysis. E–H) RAW264.7 macrophages were analyzed for iNOS and Arg‐1 gene expression that interacting with hydrogel‐modified titanium substrates were determined through immunofluorescent visualization and subsequent quantitative analysis. I,J) Analysis of the transcriptional patterns of genes associated with inflammatory responses in Raw264.7 cells cultured in the presence of titanium sheets coated with various hydrogel components, including M1‐type markers (CCR7, iNOS, IL‐1β) and M2‐type markers (CD206, Arg‐1, IL‐10).(*n =* 3; data are expressed as mean ± SD. ^*^ *p* < 0.05, ^**^ *p* < 0.01, ^***^ *p* < 0.001, ns indicates no significant difference).

In vitro evaluation confirmed that the Ti‐Lap‐C/GMA@SeNPs coating possessed pronounced abilities to exert antioxidant and anti‐inflammatory effects. Exposure to H_2_O_2_‐induced oxidative conditions demonstrated, through DCFH‐DA fluorescence imaging, a substantial reduction in intracellular ROS within MC3T3 cells. Further quantitative analysis clarified the protective characteristics of the hydrogel coating on MC3T3 cells (**Figure** **6**A,B). Flow cytometry analysis based on the DCFH‐DA assay indicated that both Ti‐C/GMA@SeNPs and Ti‐Lap‐C/GMA@SeNPs effectively alleviated oxidative stress in MC3T3 cells, a finding confirmed by additional quantitative analysis (Figure 6C,D). To evaluate the ROS‐scavenging capability of the hydrogel coatings, both DPPH radical scavenging activity and cellular oxidative stress–related protein expression were assessed. The DPPH assay revealed that integrating SeNPs markedly improved the hydrogels' ability to scavenge free radicals, with the Lap‐C/GMA@SeNPs group displaying the highest efficiency among all formulations. This result indicates that SeNPs effectively impart intrinsic antioxidative functionality to the hydrogel matrix (Figure S5A,B, Supporting Information). To further validate the antioxidative potential at the cellular level, western blotting was carried out to assess the expression levels of critical antioxidant enzymes (SOD1, SOD2, HO‐1) in MC3T3 cells cultured on hydrogel‐coated titanium substrates (Figure S5C,D, Supporting Information). Consistent with the DPPH findings, both the Ti‐C/GMA@SeNPs and Ti‐Lap‐C/GMA@SeNPs groups showed significantly upregulated expression levels of these antioxidant proteins compared to the control and Ti‐C/GMA groups. These data suggest that SeNPs not only enable direct exogenous ROS scavenging through their inherent redox activity but also potentiate endogenous antioxidant defenses by activating intracellular antioxidant signaling pathways.

After implantation, the immune microenvironment is primarily shaped by macrophage activity. When these cells are skewed toward an M1‐dominant phenotype, they trigger excessive oxidative reactions and amplify inflammatory cascades at the implant interface. In this study, the in vitro impact of Ti‐Lap‐C/GMA@SeNPs on RAW264.7 macrophages was examined. The expression of associated inflammatory markers was assessed via immunofluorescence staining and Q‐PCR. Immunofluorescence analysis showed reduced iNOS expression and increased Arg‐1 levels in macrophages exposed to Ti‐Lap‐C/GMA@SeNPs, demonstrating a tendency toward anti‐inflammatory cellular behavior. The WB results further confirmed the same conclusion (Figure S6, Supporting Information). Collectively, these observations suggest that the hydrogel modulates the immune microenvironment by attenuating pro‐inflammatory responses and guiding macrophage polarization from the M1 to the reparative M2 phenotype, demonstrating a remarkable immunoregulatory potential of the system. (Figure 6E–H). In Q‐PCR analysis, compared to the control group, the LPS‐stimulated group exhibited effective inhibition of genes associated with the M1 phenotype (CCR7, iNOS, IL‐1β) were downregulated, while those linked to the M2 phenotype (CD206, Arg‐1, IL‐10) were upregulated (Figure 6I,J). Since excessive ROS accumulation and an imbalance between M1 and M2 macrophage polarization can create a self‐perpetuating cycle—central to microenvironmental disruption—it is clear that this hydrogel coating addresses a major challenge in tissue regeneration by establishing a favorable microenvironment for efficient bone defect healing. This approach may offer a valuable avenue for future development of implant surface coatings.

### 2.5 Assessment of the Hydrogel Coating's In Vitro Osteogenic Potential

**Figure 7:**
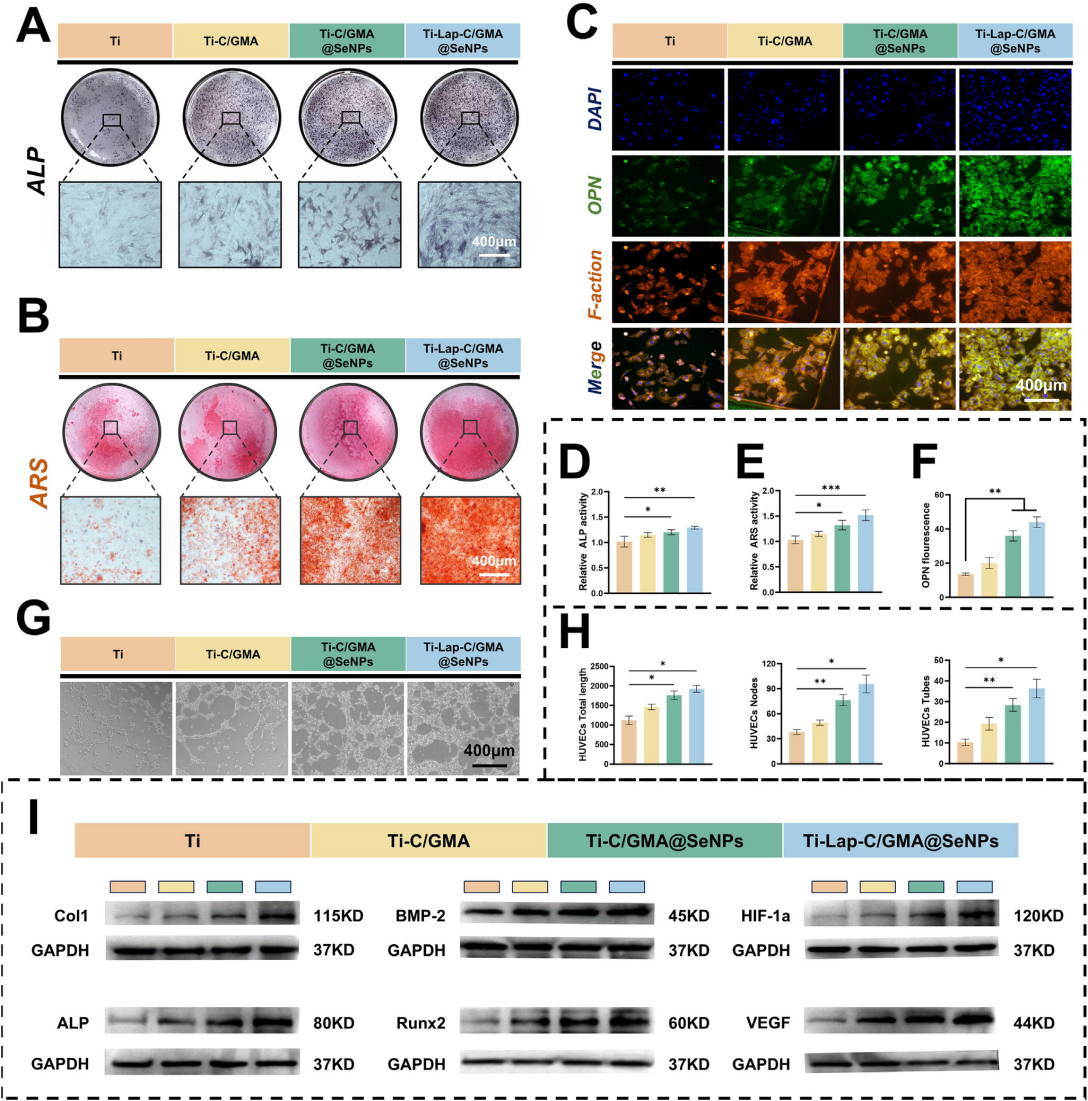
In vitro evaluation of the osteogenic performance of hydrogel‐coated titanium. A,B) ALP staining was performed on BMSCs at day 7 of induction, followed by alizarin red staining at day 14 to assess mineralized nodule formation. C) Immunofluorescence staining images of OPN associated with osteogenesis in MC3T3 cells co‐cultured with titanium plates coated with different hydrogel components. D–F) Assessment of alkaline phosphatase activity levels observed after a 7‐day culture period, matrix mineralization on day 14, and quantification of OPN immunofluorescence staining. G) Effects of titanium plates with various hydrogel coating components on tube formation of HUVECs. H) Quantitative assessment of angiogenic network formation by HUVECs, characterized by measurements of total tube length, junction density, and tubular structures. I) Protein expression patterns of BMSCs and HUVECs after a 7‐day co‐culture on hydrogel‐functionalized titanium substrates, examined by Western blot analysis. (*n =* 3; data are expressed as mean ± SD. ^*^ *p* < 0.05, ^**^ *p* < 0.01, ^***^ *p* < 0.001, ns indicates no significant difference).

To evaluate the osteogenic capacity of BMSCs induced by Ti‐Lap‐C/GMA@SeNPs, ALP and ARS staining, accompanied by quantitative analysis, were employed to characterize osteogenic differentiation and mineral matrix formation in the samples. The findings showed that ALP activity in BMSCs was significantly enhanced in the Ti‐C/GMA@SeNPs and Ti‐Lap‐C/GMA@SeNPs groups at day 7, as well as extracellular matrix mineralization on day 21, displayed statistically higher values than those observed in the control group. Furthermore, BMSCs in the Ti‐C/GMA@SeNPs group exhibited a notably higher level of osteogenic differentiation than those in the Ti‐C/GMA group, supporting the role of SeNPs in promoting osteogenesis. (**Figure** **7**A,B,D,E). For example, Chen et al. reported that SeNPs not only suppressed inflammation but also promoted osteoblast differentiation, enhanced mineralization capacity, and upregulated critical molecular indicators reflecting osteogenic lineage commitment (Runx2, ALP, OCN, and BMP2).^[^ ^41^ ^]^The immunofluorescence results of MC3T3 in the Ti‐C/GMA@SeNPs and Ti‐Lap‐C/GMA@SeNPs treatment groups demonstrated that both coatings significantly promoted osteogenic mineralization of MC3T3 cells adhered to the hydrogel coating, consistent with the previous ALP staining and ARS staining results (Figure 7C,F). As demonstrated by (Figure S8, Supporting Information), a marked upregulation of osteogenic marker mRNAs, including ALP, Runx2, OCN, and OPN, was observed in all hydrogel‐coated groups relative to the uncoated Ti group, suggesting a significant promotion of osteogenic differentiation. Notably, the Ti‐Lap‐C/GMA@SeNPs group exhibited the most pronounced increase in osteogenic gene expression, particularly in Runx2 and OCN—key transcriptional regulators and late‐stage markers of osteogenesis, respectively. The significant enhancement observed in the Lap‐C/GMA@SeNPs group is likely due to the combined actions of Lap and SeNPs, which cooperatively stimulate osteogenic differentiation via multiple signaling pathways. In particular, Lap has been shown to activate the PI3K/Akt signaling pathway, thereby facilitating osteoblast differentiation and matrix mineralization, while SeNPs help modulate redox homeostasis and enhance cell proliferation under oxidative stress. Collectively, these findings demonstrate that incorporating Lap and SeNPs into the C/GMA hydrogel coating substantially enhances the osteogenic potential of titanium implants. Importantly, this effect is confirmed in human‐derived hMSCs, highlighting the translational relevance of the coating system for clinical applications in bone regeneration.

The angiogenic potential of HUVECs cultured on hydrogel‐coated titanium substrates was assessed through a tube‐formation assay. Tubular lumen‐like structures in HUVECs were observed in real‐time in vitro at approximately 6 h, followed by quantitative analysis of the tubular lattice, including measurements of tubular network formation such as the number of closed loops, branching nodes, and cumulative tube length. The results indicated that Ti alone exhibited limited tube‐forming ability during the early stage (6 h), whereas Ti‐C/GMA@SeNPs and Ti‐Lap‐C/GMA@SeNPs showed more pronounced inducing capabilities (Figure 7G,H, Supporting Information). In this study, the expression patterns of key osteogenic proteins (BMP2, ALP, RUNX2, Col1) in BMSCs exposed to Ti, Ti‐C/GMA, and Ti‐C/GMA@SeNPs were evaluated via Western blot analysis, and Ti‐Lap‐C/GMA@SeNPs. Both Ti‐C/GMA@SeNPs and Ti‐Lap‐C/GMA@SeNPs treatment groups exhibited similar osteogenic‐promoting activity as observed previously. Additionally, Ti–Lap‐C/GMA@SeNPs group exhibited a marked elevation in the levels of proteins (Hif‐1α, VEGF) associated with pro‐angiogenic activity in HUVECs. (Figure 7I). Collectively, these findings suggest that SeNPs and Lap released from Ti‐Lap‐C/GMA@SeNPs synergistically, the treatment effectively upregulated osteogenesis‐related markers, indicating enhanced differentiation of BMSCs and MC3T3 toward the osteoblastic lineage. Quantitative evaluation of the Western blot bands is illustrated in (Figure S7, Supporting Information), highlighting the relative expression levels of the corresponding proteins.

### 2.6 Evaluation of the In Vivo Osteogenic Activity of Hydrogel Coating

**Figure 8:**
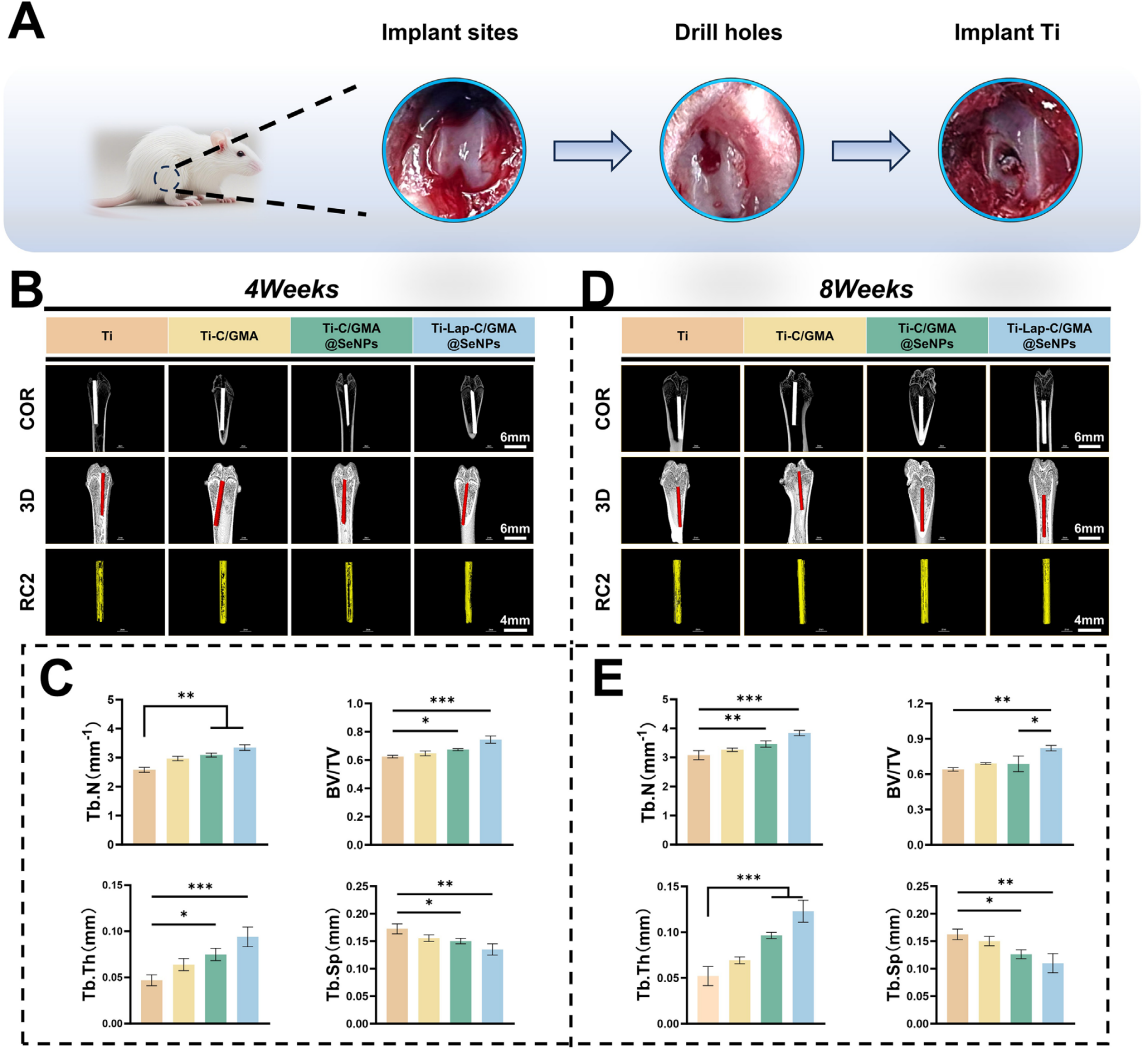
Evaluation of the in vivo osteogenic performance of hydrogel‐coated implants. A) Schematic illustration of the establishment of a mouse implant‐related distal femoral bone defect model and the corresponding treatment strategy. B,C) Micro‐CT images of the femoral defect area in each group 4 weeks post‐implantation of hydrogel‐coated implants, along with associated parameters including trabecular number (Tb.N), bone volume fraction (BV/TV), trabecular thickness (Tb.Th), and trabecular separation (Tb.Sp). D,E) Micro‐CT images of the femoral defect area in each group 8 weeks post‐implantation of hydrogel‐coated implants, along with associated parameters. (*n =* 5; data are expressed as mean ± SD. ^*^ *p* < 0.05, ^**^ *p* < 0.01, ^***^ *p* < 0.001, ns indicates no significant difference).

Given the excellent in vitro osteogenic performance of the Lap‐C/GMA@SeNPs hydrogel coating, we further evaluated its in vivo osteogenic potential using a mouse model with implant‐related distal femoral bone defects. The surgical procedure (**Figure** **8**A) involved material implantation, followed by animal sacrifice at 4 and 8 weeks post‐operation. New bone formation around the implants in the distal femoral bone defect model was evaluated using micro‐CT analysis. 3D reconstruction and quantification results revealed that minimal bone regeneration was detected in the control group at 4 weeks following surgery. In contrast, both the Ti‐C/GMA@SeNPs and Ti‐Lap‐C/GMA@SeNPs groups demonstrated bone defect repair, with significantly enhanced bone regeneration observed in the Ti‐Lap‐C/GMA@SeNPs group (Figure 8B,C). At 8 weeks post‐operation, all groups showed evidence of new bone formation; however, the Ti‐Lap‐C/GMA@SeNPs group exhibited markedly superior bone regeneration, characterized by robust bone bridging. Additionally, quantitative measurements of maximum mineralized bone volume indicated that the Ti‐Lap‐C/GMA@SeNPs group exhibited markedly increased Tb.N, Tb.Th, and BV/TV relative to the control group, whereas its Tb.Sp was significantly reduced (Figure 8D,E).

**Figure 9:**
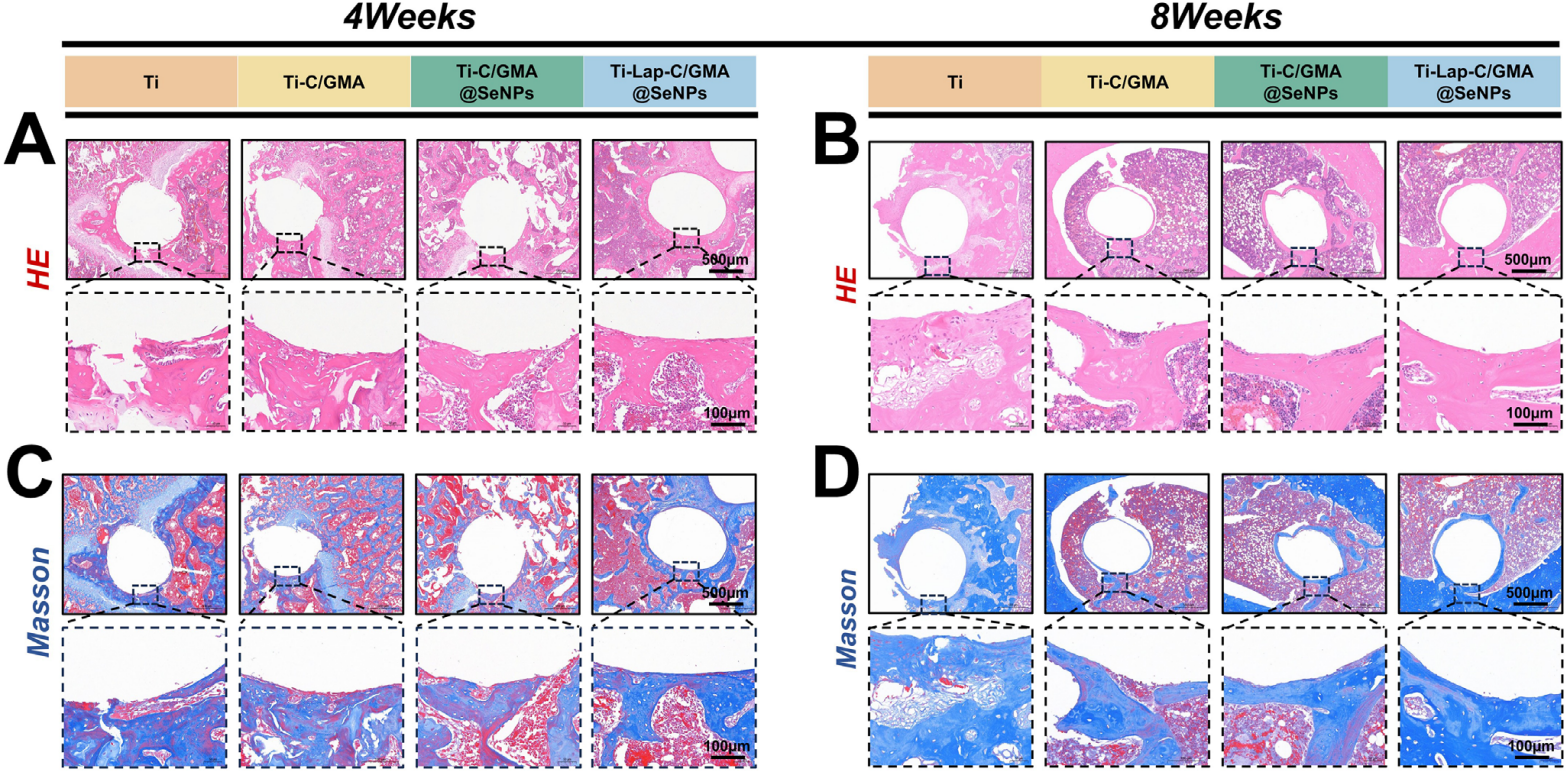
Evaluation of the in vivo osteogenic performance of hydrogel‐coated implants. A,B) HE staining images of hydrogel‐coated implants at 4 and 8 weeks post‐implantation. C,D) Masson staining images of hydrogel‐coated implants at 4 and 8 weeks post‐implantation. (*n =* 5).

Histological evaluation of newly formed bone was conducted through H&E and Masson's trichrome staining. At 4 weeks post‐surgery, the control group exhibited extensive inflammatory cell infiltration around the implants with no evident signs of new bone formation. The Ti‐C/GMA group also demonstrated limited new bone regeneration and showed inflammatory tissue infiltration in the surrounding area. In contrast, new osseous tissue was readily detectable in the sections of both the Ti‐C/GMA@SeNPs group and the Ti‐Lap‐C/GMA@SeNPs group (**Figure** **9**A). At 8 weeks post‐surgery, the new bone tissue surrounding the titanium rods in the Ti‐Lap‐C/GMA@SeNPs group was significantly thicker compared to other groups. While the Ti‐C/GMA@SeNPs group and the Ti‐C/GMA group exhibited greater bone tissue regeneration compared to the control group, their bone formation remained less pronounced compared to the Ti‐Lap‐C/GMA@SeNPs group. These findings were consistent with the H&E staining results (Figure 9B). A similar pattern was observed in the Masson's trichrome staining, where the control group predominantly exhibited mature bone tissue (appearing red), likely as a result of infection and inflammatory responses. In contrast, the Ti‐Lap‐C/GMA@SeNPs group displayed a well‐developed collagen fiber network and a pronounced increase in the formation of blue‐stained newly developed bone tissue. This group exhibited markedly elevated expression levels relative to the control group, suggesting a further elevation in osteogenic activity and highlighting the potential of this coating to promote superior bone integration. (Figure 9C,D).

**Figure 10:**
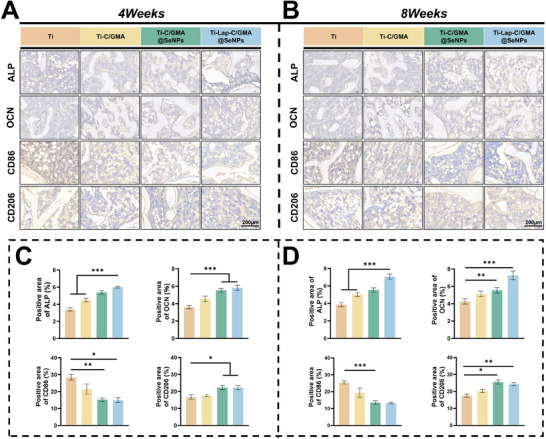
Comprehensive in vivo analysis of bone‐forming potential and immune‐modulating behavior of hydrogel‐modified titanium implants. A,B) Immunohistochemical staining images of osteogenesis‐related markers (ALP, OCN) and inflammation‐related markers (CD86, CD206) at 4 and 8 weeks post‐implantation of hydrogel‐coated implants. C,D) Quantitative analysis of osteogenesis‐related markers (ALP, OCN) and inflammation‐related markers (CD86, CD206) by immunohistochemistry at 4 and 8 weeks post‐implantation of hydrogel‐coated implants. (*n =* 5; data are expressed as mean ± SD. ^*^ *p* < 0.05, ^**^ *p* < 0.01, ^***^ *p* < 0.001, ns indicates no significant difference).

Immunohistochemical (IHC) staining was conducted at 4 and 8 weeks post‐surgery to assess ALP and OCN expression levels—two key osteogenic transcription factors—in the bone defect region. Quantitative evaluation demonstrated a notable enhancement in ALP and OCN levels in the Ti‐C/GMA@SeNPs and Ti‐Lap‐C/GMA@SeNPs samples compared with those in the control group, demonstrating enhanced osteogenic activity in these treatment groups. In contrast, only a few positive regions were observed in the Ti‐C/GMA and control groups. Furthermore, the key inflammatory markers CD86 and CD206 were examined. The pro‐inflammatory marker CD86 showed a marked reduction in the Ti‐C/GMA@SeNPs and Ti‐Lap‐C/GMA@SeNPs groups at both 4 and 8 weeks compared to the control group, while the marker CD206 associated with anti‐inflammatory activity exhibited higher positive areas in these groups than in the control group. Within the same groups, the anti‐inflammatory performance at 8 weeks was superior to that at 4 weeks, indicating sustained long‐term release of anti‐inflammatory factors from Ti‐Lap‐C/GMA@SeNPs. Collectively, these findings demonstrate that the synergistic effects of SeNPs and Lap enhance bone regeneration in vivo, aligning well with the earlier in vitro findings (**Figure** **10**).

**Figure 11:**
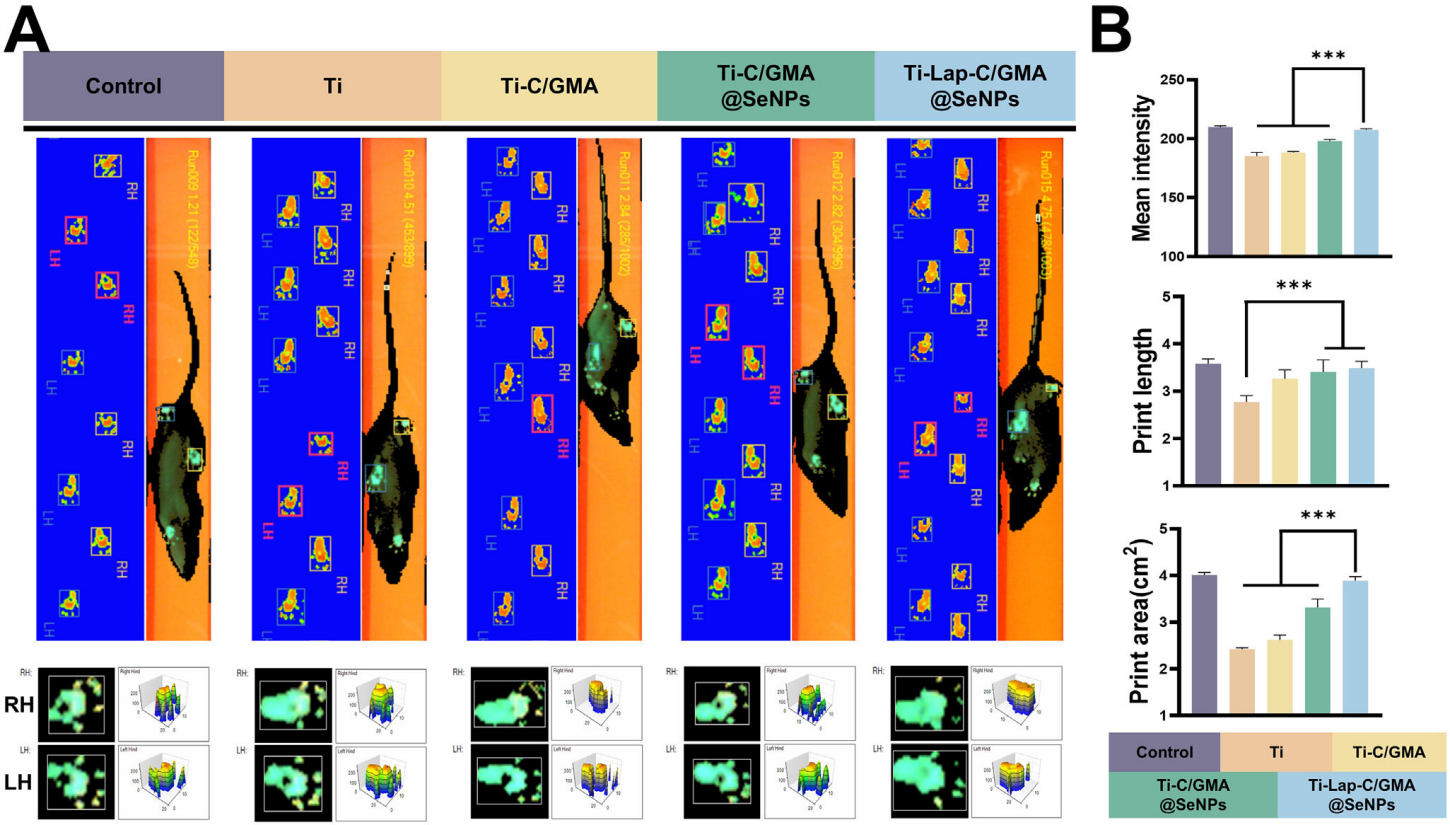
Evaluation of gait patterns in rats eight weeks after implantation. A) Representative footprints showing gait morphology, stride length, and force distribution; B) Quantitative evaluation of footprint characteristics and mean pressure intensity. (*n =* 5; data are expressed as mean ± SD. ^*^ *p* < 0.05, ^**^ *p* < 0.01, ^***^ *p* < 0.001, ns indicates no significant difference).

Furthermore, the 8‐week rat paw print analysis further validated the efficacy of Ti‐Lap‐C/GMA@SeNPs in repairing distal femoral bone defects (Figure 11A). As indicated by the quantitative data, the values obtained for the experimental groups were higher than those of the control and Ti‐C/GMA groups, the Ti‐C/GMA@SeNPs group and the Ti‐Lap‐C/GMA@SeNPs group exhibited significant improvements in average forelimb contact area, average forelimb pressure, and stride length, suggesting the partial recovery of knee joint function in rats (**Figure** **11B**). Nevertheless, these parameters did not fully match the performance levels observed in the Control group  .

### 2.7 Subcutaneous Degradation Model of the Hydrogel Coating

All hydrogel formulations exhibited progressive degradation over time, without any statistically significant variation in residual body weight across the different groups. Histological analysis using H&E staining of the surrounding tissues at both time points revealed mild inflammatory responses and the absence of adverse tissue reactions, indicating favorable biocompatibility. These findings suggest that the hydrogels underwent stable in vivo degradation without inducing significant tissue damage or fibrotic encapsulation (Figure S9, Supporting Information).

### 2.8 *In* *Vivo* Evaluation of Systemic Toxicity Induced by Hydrogel Coating

Following 8 weeks of implantation with Ti‐Lap‐C/GMA@SeNPs, specimens from the heart, liver, spleen, lungs, and kidneys were harvested for analysis and subjected to H&E staining for histopathological evaluation. (Figure S10, Supporting Information). The findings demonstrate that Ti‐Lap‐C/GMA@SeNPs possess outstanding biocompatibility and do not induce toxicity in vital organs. In detail, cardiac tissue exhibited preserved myocardial organization with no detectable edema or inflammatory cell infiltration. Liver samples revealed intact hepatic lobular architecture, absence of hepatocellular degeneration, and no presence of inflammatory cells. Lung sections maintained normal alveolar morphology, characterized by clear air spaces, undamaged alveolar walls, and no indications of inflammation, edema, or hemorrhage. Likewise, kidney tissues displayed healthy glomeruli and renal tubules with no pathological features such as necrosis or fibrotic changes. Collectively, these findings, in conjunction with the in vitro cytotoxicity study, verify the excellent biocompatibility of Ti‐Lap‐C/GMA@SeNPs, underscoring their potential as a highly suitable candidate material for bone regeneration applications.

## 3 Discussion

Fracture healing involves an extended and intricate biological process that depends on intricate biological and immune mechanisms, typically involving early inflammation, mid‐term bone repair, and long‐term bone remodeling. In the early stage of bone injury, the microenvironment accumulates excessive ROS, while macrophages phagocytose cell debris, playing a pivotal role in orchestrating inflammatory cascades and enhancing the levels of pivotal cytokines, including IL‐1β, TNF‐α, and IL‐6.^[^ ^42^ ^]^ as the healing process progresses into the bone repair phase, tissue regeneration requires the inflammatory milieu to evolve from a pro‐inflammatory state to an anti‐inflammatory one. During this phase, mesenchymal stem cells (MSCs) along with multiple progenitor cell types are recruited to the site of injury, beginning their differentiation into osteoblasts—crucial cells responsible for new bone synthesis. In parallel, endothelial cells drive angiogenesis, promoting angiogenesis, which is essential for delivering oxygen and nutrients to the developing bone tissue. The coordinated interaction between osteogenesis and angiogenesis is critical for successful bone regeneration and functional restoration.^[^ ^43^, ^44^ ^]^ Additionally, bacterial infection remains one of the most significant risk factors contributing to delayed healing or nonunion of fractures. Infections not only prolong inflammation but also compromise the integration of titanium implants with surrounding bone tissue—a process known as osseointegration.^[^ ^45^ ^]^ Severe bacterial infections and inadequate osseointegration around titanium implants can lead to surgical failure and pose a serious threat to patient health.^[^ ^46^, ^47^ ^]^ Given these challenges, there is a growing demand for dual‐functional implants capable of both antibacterial activity and osseointegration. However, achieving both properties simultaneously remains challenging. Traditional titanium implants, despite being commonly employed for their advantageous mechanical properties and biocompatibility, lack intrinsic antibacterial capabilities and have limited bioactivity to actively promote bone healing. To address these limitations, researchers have focused on surface modifications of titanium implants to enhance their functionality.^[^ ^3^, ^13^ ^]^ For instance, some researchers have attached antimicrobial peptides to the surface of titanium implants to combat bacterial infections,^[^ ^48^, ^49^ ^]^while others have coated titanium implants with biomaterials that promote bone regeneration to enhance bone healing.^[^ ^50^ ^]^

In this work, we engineered a composite hydrogel coating incorporating Lap and SeNPs, cross‐linked by CMCSMA and GelMA. The composite hydrogel formed by the mixture of CMCSMA and GelMA exhibits excellent biocompatibility,^[^ ^51^, ^52^ ^]^ promoting cell adhesion, proliferation, and differentiation while conferring certain antibacterial activity.^[^ ^21^, ^22^ ^]^ The composite hydrogel employed in this study exhibited a progressive reduction in both volume and mass over a 4‐week period in vivo. Notably, no significant inflammatory or fibrotic responses were detected in the surrounding tissues. These findings further validate the favorable tissue compatibility and consistent biodegradation profile of the hydrogel system under physiological conditions. The incorporation of SeNPs effectively scavenges excessive ROS in the bone repair microenvironment, in accordance with the mechanistic pathway that has been previously reported for SeNPs protecting bone cells from oxidative stress‐induced damage.^[^ ^53^ ^]^ This efficient ROS clearance further alleviates cellular inflammatory responses, inhibits macrophage polarization toward the M1 phenotype, and promotes transition to the M2 phenotype, thus demonstrating immunomodulatory capabilities. Since excessive ROS and an imbalance between M1 and M2 macrophages can trigger a self‐perpetuating cycle that significantly contributes to disruption of the local microenvironment, the sustained release of SeNPs in the Lap‐containing composite hydrogel demonstrates superior performance compared to simple hydrogels. In vitro studies revealed that Mg^2^⁺ released from Lap not only synergizes with SeNPs to disrupt bacterial membrane potential, exerting antibacterial effects against *E. coli* and *S. aureus*,^[^ ^27^, ^54^, ^55^ ^]^ but also enhances the adhesion of mouse pre‐osteoblasts (MC3T3) and HUVECs on the hydrogel coating. Furthermore, Mg^2^⁺ activates the PI3K‐Akt signaling pathway, promoting osteogenic differentiation of BMSCs.^[^ ^35^, ^36^ ^]^ The bone integration‐promoting effect is attributed to the temporally controlled sequential release of SeNPs and Mg^2^⁺ and their synergistic actions. Early released SeNPs rapidly scavenge ROS, reduce oxidative stress, and protect cells and matrix. They also exert antibacterial effects, prevent biofilm formation, and lower infection risk. SeNPs modulate acute inflammation by inhibiting M1 macrophages and promoting pro‐reparative immune states. Sustained Mg^2^⁺ release later supports immune homeostasis, reduces chronic inflammation, and enhances osteogenic signaling for accelerated osseointegration. This engineered hydrogel coating effectively addresses the long‐standing challenges associated with bone tissue repair by establishing a bioactive interface that promotes osteoblast adhesion, activation, and coordinated matrix regeneration. Notably, the Mg^2^⁺ and SiO_3_^2^^−^ ions released from Lap significantly promote vascular endothelial cell proliferation and differentiation, facilitating bone formation. In vivo studies demonstrated that titanium rods modified with the composite hydrogel and implanted into the distal femur of rats exhibited significantly increased new bone formation and markedly reduced inflammatory cell infiltration around the titanium rods, as confirmed by micro‐CT and histological analyses, further validating the mineralization‐promoting ability of the composite coating.

Although this coating exhibits multifunctional advantages, its clinical translation still faces several challenges. First, the potential cytotoxicity of SeNPs after long‐term implantation requires further validation using larger animal models (e.g., dogs or sheep) and extended follow‐up periods. Second, in commercial‐scale production, ensuring the uniformity and consistency of the coating poses significant technical difficulties. The release kinetics of SeNPs can be effectively modulated by tuning the crosslinking density of GelMA and CMCSMA, thereby minimizing the risk of high‐dose toxicity associated with rapid release during the initial phase. Alternatively, the incorporation of chemical interactions—such as ionic or hydrogen bonding—or the integration of natural extracts (e.g., silk protein, pectin) and synthetic polymeric matrices (e.g., PEG‐PLA block copolymers) may further refine the release profile.^[^ ^56^, ^57^ ^]^ Nevertheless, as previously discussed, the early‐stage release of SeNPs also demonstrates therapeutic potential by mitigating acute inflammatory responses and ROS accumulation following implantation, indicating a certain degree of biological compatibility.

## 4 Conclusion

This study proposed a coating modification strategy to address the imbalance of the peri‐implant microenvironment. The strategy involves a composite hydrogel coating composed of CMCSMA and GelMA, loaded with Lap and SeNPs. In vitro experiments demonstrated that this coating exhibits excellent antibacterial properties, promotes cell adhesion, eliminates excessive intracellular ROS, alleviates inflammatory responses, and enhances mineralization and osteogenic capacity. Furthermore, its bone repair activity was verified in a rat model. These results indicate that this composite hydrogel coating could serve as a potential alternative for modifying titanium implants in future clinical applications.

## 5 Experimental Section

### Materials

Commercially available native titanium foil (0.1 mm thick, 99.5% purity) and titanium rods (1.0 mm in diameter, 10.0 mm in length) were obtained through the Northwest Institute for Nonferrous Metal Research (Xi'an, China). The titanium foils were sectioned into 10 mm × 10 mm pieces to match the dimensions of 24‐well and 6‐well culture plates for use in in vitro studies. Carboxymethyl chitosan (CMCS), gelatin (Gel), methacrylic anhydride (MA), and laponite (Lap) were supplied by Macklin (Shanghai, China). The SeNPs stock solution was kindly provided by the Institute of Nano Science, Soochow University (Suzhou, China). Minimum essential medium‐α (MEMα), fetal bovine serum (FBS), and phosphate‐buffered saline (PBS) used for cell culture were supplied by Gibco (Carlsbad, CA, USA). Other analytical‐grade reagents and assay kits were sourced from standard laboratory vendors.

### Methods—Preparation of CMCSMA

In brief, a 3 wt.% solution of CMCS was prepared in distilled water. Then, 7% (v/v) methacrylic anhydride (MA) was introduced into the solution, and the pH was maintained at 8.0 with 5 M NaOH. The reaction mixture was stirred continuously for 12 h in an ice‐cooled environment to ensure proper modification. Afterward, the product was dialyzed against distilled water for three days at room temperature using a dialysis membrane with a molecular weight cutoff of 8–14 kDa. The purified product was filtered and freeze‐dried to obtain CMCSMA, which was maintained at −20 °C before further applications.

### Methods—Preparation of GelMA

Briefly, gelatin was dissolved in phosphate‐buffered saline (PBS) at 50 °C to prepare a 10% (w/v) solution, with continuous stirring until fully solubilized. To obtain a final concentration of 4% (v/v), methacrylic anhydride (MA) was introduced slowly and carefully under controlled conditions, followed by intense stirring at 50 °C for 3 h to promote functional group incorporation. The reaction was terminated by adding five times the volume of pre‐warmed PBS (50 °C). The mixture was subsequently dialyzed against distilled water at 40 °C for 72 h using a dialysis membrane with a molecular weight cutoff of 8–14 kDa. Upon completion of dialysis, the solution was subjected to freezing and freeze‐drying processes to obtain GelMA, which was stored at −20 °C before being used in later experiments.

### Methods—Fabrication of Ti Implants

The native titanium foil (thickness: 0.1 mm, purity: 99.5%) and titanium rods (diameter: 1.0 mm, length: 10 mm) were polished and ultrasonically cleaned. CMCSMA was dissolved in a mixture of deionized water and photoinitiator (7:3, *v/v*) to prepare a 10% (*w/v*) solution, while GelMA was dissolved in the same solvent system to prepare an 8% (*w/v*) solution. The two solutions were subsequently mixed in a 1:1 ratio (*v/v*), and different concentrations of SeNPs and Lap were selectively added to each component. The mixtures were then thoroughly homogenized by ultrasonication for subsequent experiments. The titanium rods and titanium plates were immersed in the hydrogel solutions for 5 min, removed and subjected to ultraviolet irradiation for 10 s to ensure full crosslinking of the hydrogel network. The samples were then dried at 37 °C. Finally, the treated titanium rods and plates were individually sealed and subjected to ultraviolet disinfection for 35 min before being stored for further use (Figure S2, Supporting Information).

### Characterization—Characterization of Selenium Nanomaterials

The morphological characteristics of SeNPs were examined via transmission electron microscopy (TEM) using a Hitachi TEM system (Japan) operated at an accelerating voltage of 80.0 kV in high‐contrast (HC) mode, with images captured at a magnification of 1 × 10⁶. Furthermore, the size distribution of the SeNPs was determined at ambient temperature using a Nano‐ZS analyzer (Malvern, Worcestershire, UK).

### Characterization—Characterization of Titanium Implants

The surface morphology of titanium alloy plates following various treatments was evaluated by scanning electron microscopy (SEM) using a Thermo Scientific Apreo 2C instrument (Waltham, MA, USA), performed ≈60 s after sputter‐coating with gold. Imaging was conducted at an accelerating voltage of 3.0 kV. Furthermore, energy‐dispersive X‐ray spectroscopy (EDS) was employed to analyze the elemental composition and distribution across the nanomaterials.

### Characterization—Hydrogel Characterization

The molecular configurations of CMCSMA and GelMA were confirmed by recording their ^1^H NMR spectra using proton nuclear magnetic resonance spectroscopy. For this analysis, 20 mg of each sample was dissolved in deuterium oxide (D_2_O) and measured using a Bruker AVANCE‐600 spectrometer (600 MHz, Bruker, Germany).

The rheological behavior and compressive properties of the C/GMA, C/GMA@SeNPs, and Lap‐C/GMA@SeNPs hydrogels were assessed using a rotational rheometer (Kinexus Lab+, NETZSCH, Germany) fitted with a 20 mm parallel‐plate configuration. All measurements were performed at 25 °C with a constant plate gap of 1 mm. Prior to testing, the hydrogels were allowed to equilibrate for 10 min to ensure structural stability and minimize residual stresses.

Rheological analysis was carried out by first performing strain sweep measurements at a constant frequency of 1 Hz, with the strain amplitude varied from 0.1% to 1000% to determine the linear viscoelastic region (LVR). Following this, frequency sweep tests were conducted within the LVR using a strain of 1%, across a frequency range of 0.1–100 Hz, to evaluate the storage modulus (G′) and loss modulus (G″). The viscoelastic behavior of each hydrogel formulation was examined to investigate how the addition of SeNPs and the inclusion of Laponite influence crosslinking density and the structural stability of the network.

Compressive mechanical tests were carried out using the same rheometer in compression mode. Cylindrical hydrogel samples (10 mm in diameter and 5 mm in height) were compressed at a constant strain rate of 1 mm·min^−1^ until achieving 80% deformation relative to their original height. Stress–strain curves were recorded in real time, and the compressive modulus was derived from the linear elastic region, typically corresponding to 5–15% strain. All experimental procedures were repeated on three separate occasions to confirm the consistency and repeatability of the results.

The swelling behavior of the hydrogels was evaluated by first measuring and recording the initial weight of each hydrogel sample as W_0_. Each sample was immersed in PBS (pH 7.4) and incubated at 37 °C to simulate physiological conditions. The weight change of the specimens was recorded at predefined time points to evaluate degradation kinetics. The swelling ratio was determined using Equation (1), where W_t_ refers to the measured hydrogel mass recorded at each designated time interval.

(1) $$Swelling\;ratio\;\%\;=\frac{Wt-W0}{W0}\times 100\%$$

The degradation profile of the hydrogels was evaluated over a 30‐day period by monitoring weight loss in PBS at 37 °C. At specified time intervals, samples were retrieved, dried to constant weight, and their residual dry masses were measured. The pre‐incubated PBS was subsequently exchanged for a freshly prepared buffer supplemented with 3 mg mL^−1^ collagenase I to enable continuous enzymatic digestion. The degradation percentage was determined using Equation (2), where Wd_0_ represents the initial dry weight of each hydrogel group, and Wdt indicates the dry weight measured at each respective time point after incubation.

(2) $$Degradation\;\%\;=\frac{Wd0-Wdt}{Wd0}\times 100\%$$

### Characterization—Ion Release Performance Test

The hydrogel was fabricated into disc‐shaped specimens with a diameter of 9 mm and a thickness of 3 mm. Subsequently, the samples were incubated in PBS on a shaker at 37 °C under constant agitation conditions. At predetermined time intervals, the supernatant was collected, and the concentrations of Se and Mg^2+^ were quantified using ICP‐MS.

### Cell Culture and Cytocompatibility Assessment

Rat‐originated bone marrow mesenchymal stem cells (BMSCs) and the murine macrophage cell line RAW264.7 were selected for subsequent in vitro evaluations, and MC3T3‐E1 preosteoblast cells, HUVECs used for cell culture experiments were purchased from OriCell Biotechnology Co., Ltd. (Guangzhou, China).All cell lines were cultured in MEMα medium (Gibco, USA) enriched with 10% fetal bovine serum and 1% penicillin–streptomycin under standard incubator conditions. Cell proliferation in response to different hydrogel compositions was quantified by the CCK‐8 colorimetric assay.

### Cell Culture and Cytocompatibility Assessment—Biocompatibility Assessment of SeNPs

First, SeNPs solutions at different concentrations (5, 10, 30, and 50 µg mL^−1^) were obtained by diluting the SeNPs stock solution in deionized water. Subsequently, hydrogels loaded with these SeNPs concentrations were co‐cultured with MC3T3 and HUVECs. The hydrogel groups were designated as C/GMA@SeNPs‐1, C/GMA @SeNPs‐2, C/GMA@SeNPs‐3, and C/GMA@SeNPs‐4, corresponding to the respective SeNPs concentrations. The optimal SeNPs concentration was established by evaluating cellular reactions to the released selenium nanoparticles.

### Cell Culture and Cytocompatibility Assessment—Biocompatibility Assessment of Lap

To prepare hydrogels with varying Lap contents, four samples were formulated with Lap concentrations of 1, 3, 5, and 10 mg mL^−1^, designated as 1‐Lap‐ C/GMA, 3‐Lap‐ C/GMA, 5‐ C/GMA, and 10‐Lap‐ C/GMA, respectively, to evaluate the cytotoxicity of Lap. Approximately 500 µL of each prepolymer solution was dispensed into the wells of a 24‐well plate and subjected to photo‐crosslinking under UV exposure. Bone marrow–derived mesenchymal stem cells (5 × 10⁴ per well) were subsequently seeded onto the hydrogel surfaces. After 1 and 3 days of incubation, the metabolic activity of the cells was quantified at 450 nm using the CCK‐8 colorimetric assay (Dojindo, Japan) in accordance with the manufacturer's protocol. Cell viability was determined using the following equation:

(3) $$\mathrm{Cell}\;\mathrm{viability}=\left(OD_{\mathrm{test}}-OD_{\mathrm{blank}}\right)/\left(OD_{\mathrm{control}}-OD_{\mathrm{blank}}\right)\times 100\%$$

The influence of the hydrogel coating on cell viability was evaluated using a Live/Dead Cell Staining Kit (Dojindo, Japan). The cultivation process was implemented as per the prior methodological description.

### Cell Culture and Cytocompatibility Assessment—Wound Healing Assay

Prior to cell seeding, horizontal alignment marks were inscribed on the base of each 6‐well culture plate. Subsequently, HUVECs (1 × 10⁶ cells per well) were cultured for 12 h at 37 °C with 5% CO_2_ to achieve uniform cell attachment. At ≈80% cell coverage, HUVEC monolayers were prepared for subsequent treatment, a longitudinal scratch was made on the cell monolayer using a 200 µl pipette tip. The cells were then washed three times with PBS to remove any floating cells. Subsequently, 2 mL of serum‐free medium and titanium discs coated with hydrogels of different compositions were added. Following 24 h incubation at 37 °C in 5% CO_2_, microscopic observation was conducted to assess the recovery of the defect regions, the quantitative evaluation of images was carried out using ImageJ software (NIH, Bethesda, MD, USA).

### Cell Culture and Cytocompatibility Assessment—Transwell Cell Invasion Assay

Following centrifugation, HUVECs were resuspended in serum‐free culture medium. A 100 µL aliquot of the suspension, containing ≈1 × 10⁴ cells, was seeded into the upper compartment of a 24‐well Transwell insert. In the lower chamber, 600 µL of culture medium that had been pre‐incubated with titanium discs coated with various hydrogel formulations was added, and the insert was carefully placed into the well. Upon completion of 24 h incubation at 37 °C under 5% CO_2_ conditions, the upper compartment underwent two sequential washes with PBS to ensure removal of residual components, and non‐migrated cells remaining on the interior surface were gently removed using a sterile cotton swab. The migrated cells on the underside of the membrane were fixed with 4% paraformaldehyde for 30 min and washed three times with PBS. To ensure accurate quantification, any residual cells on the upper side were meticulously wiped away. Subsequently, the cells that had migrated to the lower surface were stained with crystal violet for 10 min, rinsed with PBS, and visualized using a Zeiss Axio Vert.A1 inverted fluorescence microscope to assess cell migration activity.

### Cell Culture and Cytocompatibility Assessment—The Role of Hydrogel Coating in Alleviating Oxidative Stress

First, the hydrogel's capacity to mitigate elevated intracellular reactive oxygen species (ROS) was evaluated. In brief, as described earlier, cells were cultured on the hydrogel surface and subsequently exposed to a 200 µm H_2_O_2_‐containing medium for 12 h to induce oxidative stimulation. Following this oxidative stress challenge, following enzymatic detachment, cells were collected and incubated with the DCFH‐DA probe (Dojindo, Japan) for ROS labeling. Fluorescence imaging and flow‐cytometric quantification were then employed to evaluate intracellular ROS levels.

The intrinsic antioxidant potential of the hydrogel coating, indicative of its external free radical scavenging ability, was further assessed using the 2,2‐diphenyl‐1‐picrylhydrazyl (DPPH) assay. In brief, a 200 µL aliquot of the hydrogel precursor solution was applied to a substrate and crosslinked via photopolymerization to form a stable gel film. The obtained hydrogel was then immersed in 1 mL of a 0.3 mmol/L DPPH solution prepared in 96% ethanol (DPPH, MACKLIN) and kept under dark conditions for 1 h to prevent light‐induced degradation. After the incubation step, the supernatant was gently collected, and the absorbance at 517 nm was determined using a microplate spectrophotometer. The DPPH radical scavenging percentage was calculated using the following equation:

(4) $$DPPH.Scavenging\%\;=\frac{Ac-An}{Ac}\times 100\%$$

here, A_0_ was defined as the absorbance of the ethanol‐based DPPH solution measured under identical conditions but without hydrogel exposure, serving as the experimental blank, while A_n_ indicates the absorbance measured after incubation with the hydrogel sample. For accuracy validation, measurements were conducted in three independent replicates, allowing assessment of both experimental consistency and statistical reliability.

### Cell Culture and Cytocompatibility Assessment—Immunomodulatory Mechanisms Induced by the Hydrogel Coating

Moreover, differential influences of the various treatment groups on macrophage polarization were identified in this study. Specifically, each titanium plate was fixed within a 24‐well culture plate, and RAW264.7 macrophages were introduced at a density of 2 × 10⁵ cells per well for subsequent incubation. Macrophage polarization toward the M1 phenotype was stimulated by culturing the cells in medium containing 1 µg mL^−1^ lipopolysaccharide (LPS; Solarbio, Beijing, China), mimicking the inflammatory response following implant placement. After seven days of culture, the mRNA expression patterns corresponding to M1‐associated inflammatory genes (CCR7, iNOS, IL‐1β) and M2‐associated anti‐inflammatory genes (CD206, Arg‐1, IL‐10) were examined via quantitative real‐time PCR to evaluate macrophage polarization behavior. Additionally, Titanium plates from each experimental group were positioned in 6‐well plates, and 1.5 × 10⁶ Raw264.7 cells were seeded per well. To induce M1 macrophage polarization, lipopolysaccharide (LPS, 1 µg mL^−1^; Solarbio, Beijing, China) was added to the culture medium. Immunofluorescent labeling was carried out to assess the expression of iNOS and Arg‐1 in Raw264.7 cells, and fluorescent images were captured using a Zeiss Axio Vert.A1 inverted fluorescence microscope for morphological and phenotypic analysis.

### Cell Culture and Cytocompatibility Assessment—ALP Staining and ARS Staining

To initiate cell culture, BMSCs were inoculated into 24‐well plates at a seeding density of 1 × 10⁵ cells per well, cultured in direct contact with titanium substrates coated with hydrogels of varying compositions. The cells were cultured in osteogenic induction medium composed of αMEM supplemented with 10% FBS, 10 nm vitamin D3, 10 mM β‐glycerophosphate, 10 nm dexamethasone, 100 mg mL^−1^ streptomycin, and 100 U mL^−1^ penicillin. At days 7 and 14 post‐induction, alkaline phosphatase (ALP) expression was assessed using the BCIP/NBT ALP Staining Kit (Beyotime Biotechnology, China), while mineralized nodule formation was evaluated through ARS staining with ARS Solution (LEAGENE, China). Additionally, ALP activity was quantitatively measured using the ALP Activity Assay Kit (Beyotime Biotechnology Institute, China) according to the manufacturer's instructions.

### Cell Culture and Cytocompatibility Assessment—OPN Immunofluorescence Labeling

For cell culture experiments, MC3T3 cells were inoculated at a seeding density of 1 × 10⁵ cells per well in a 24‐well plate and co‐cultured with titanium sheets coated with hydrogel formulations of different compositions for 7 days. The level of the osteogenic differentiation marker OPN was assessed by immunofluorescence staining. The fluorescent signal intensity of OPN in MC3T3 cells cultured on titanium substrates was visualized using a Zeiss Axio Vert.A1 inverted fluorescence microscope, and a semi‐quantitative assessment was carried out with ImageJ software.

### Cell Culture and Cytocompatibility Assessment—Capillary Tube Formation Assay

Prior to the experiment, Matrigel (BD, USA), 96‐well plates, and pipette tips were pre‐chilled overnight at 4 °C. During the procedure, the 96‐well plates were maintained on ice to prevent premature gelation. Fifty microliters of Matrigel were loaded into 96‐well plates preconditioned at low temperature, and the mixture was then incubated at 37 °C for 30 min to initiate gelation. HUVECs were suspended in cell culture extracts that had been preconditioned with titanium substrates coated with various hydrogel formulations. Then, 50 µL of the cell suspension (containing 1 × 10⁴ cells per well, with three replicates per group) was carefully seeded onto the pre‐gelled Matrigel surface. All cell cultures were incubated under standard physiological conditions (37 °C, 5% CO_2_, humidified atmosphere), tube formation was monitored dynamically using phase‐contrast microscopy. Quantitative evaluation of angiogenic structures, including total tube length and number of branch points were quantified using ImageJ software.

### Antibacterial Assay

The effectiveness of the antimicrobial activity of the samples against Escherichia coli (*E. coli*, ATCC 8739) and Staphylococcus aureus (*S. aureus*, ATCC 6538) was assessed using the colony‐forming unit (CFU) counting method. Before testing, all samples, including hydrogel‐coated specimens, were sterilized. Fresh LB broth was inoculated with a single colony of either *E. coli* or *S. aureus* and incubated under shaking conditions (200 rpm, 37 °C) overnight. After incubation, the bacterial cultures were diluted to obtain a final concentration of 1 × 10⁶ CFU mL^−1^. The group without hydrogel served as the negative control, A gentamicin solution (4 µg·mL^−1^) was used as the positive control under identical experimental conditions. To evaluate antibacterial efficacy, the hydrogel materials were exposed to bacterial suspensions under shaking conditions (37 °C, 180 rpm) for 18 h.

To assess the antibacterial efficacy of the hydrogels visually, a live/dead bacterial viability assay was conducted using the SYTO9/PI Live and Dead Bacteria Staining Kit (Beijing BioLab Technology Co., Ltd., China). After 18 h of co‐culture, the hydrogel samples were gently washed three times with sterile PBS to remove loosely attached bacteria. The samples were then incubated with a staining solution containing 5 µm SYTO 9 and 2 µg mL^−1^ propidium iodide (PI) for 15 min in the dark at room temperature. Stained samples were subjected to PBS washing to remove remaining dye, and fluorescence acquisition was conducted with a laser scanning confocal microscope (LSM800, Zeiss, Germany). Intact, viable bacteria emitted green fluorescence due to SYTO 9 staining, while those with compromised membranes, indicating cell death, showed red fluorescence from PI uptake. The bacterial cultures treated with gentamicin and the negative control group were also photographed using the same method.

To standardize bacterial density, cultures were adjusted in Luria–Bertani medium to ≈1 × 10⁶ CFU mL^−1^ prior to use. For biofilm cultivation, 2 mL of the bacterial suspension (diluted to the required concentration) was inoculated into each well of a six‐well plate, with sterile glass coverslips prearranged at the bottom as adhesion substrates. The plates were incubated statically at 37 °C for 24 h to allow mature biofilm formation. Upon completion of incubation, the biofilms were washed twice with PBS to eliminate bacteria that did not adhere to the surface. Subsequently, hydrogel samples with varying compositions and gentamicin solution were applied to the pre‐established biofilm and co‐cultured under static conditions at 37 °C for an additional 24 h. Once the treatment was completed, the hydrogels were carefully lifted off, and the biofilms were rinsed with PBS to remove any residual planktonic cells. A working solution for fluorescence staining was prepared as per the manufacturer's protocol, and 200 µL was applied directly onto each coverslip. The samples were subsequently incubated in the dark at 37 °C for 15 min, then gently washed twice with PBS to eliminate any unbound dye. Finally, the stained biofilms were transferred onto microscope slides and examined using a laser scanning confocal microscope to generate 3D reconstructions of the biofilm structure and spatial organization.

The group without hydrogel served as the negative control. A gentamicin solution (4 µg·mL^−1^) was used as the positive control under identical experimental conditions. To evaluate antibacterial efficacy, the hydrogel materials were exposed to bacterial suspensions under shaking conditions (37 °C, 180 rpm) for 18 h. After 18 h, the bacterial suspension was serially diluted using 100 µL aliquots in sterile saline to prepare working concentrations for subsequent experiments to achieve concentrations of 10^1^, 10^2^, 10^3^, 10⁴, and 10⁵ CFU mL^−1^.Then, 200 µL of each diluted solution was inoculated onto blood agar plates and incubated at 37 ± 1 °C for 24 h, with three replicates per group. The bacterial inhibition rate was calculated using the following equation:

(5) $$\mathrm{Bacterial}\;\mathrm{survival}\;\mathrm{rate}\left(\%\right)=B/A\times 100$$

In the aforementioned formula, A and B denote the colony‐forming unit (CFU) counts of the experimental sample and the control sample on the agar plate, respectively.

The bacterial suspension was collected after the colony‐forming unit (CFU) determination and centrifuged to obtain the bacterial pellet. Subsequently, the sample was fixed with 4% formaldehyde solution at 4 °C for 4 h, followed by dehydration with a graded ethanol series and drying for SEM characterization.

### Quantitative Real‐Time Polymerase Chain Reaction (qRT‐PCR)

Human bone marrow‐derived mesenchymal stem cells (hMSCs) were obtained from Guangzhou Yanshi Technology Co., Ltd. (Guangzhou, China) and seeded onto six‐well plates (1 × 10⁶ cells per well) and co‐cultured with hydrogel‐coated titanium substrates of distinct compositions for seven days. Total RNA from hMSCs was obtained with TRIzol reagent (Sigma–Aldrich, USA) in accordance with the manufacturer's guidelines. To verify RNA quality, the absorbance at 260 nm was recorded with a NanoDrop ND‐2000 spectrophotometer (Thermo Fisher Scientific, USA), and the concentration and purity were subsequently calculated. Reverse transcription was performed using a C1000 Touch Thermal Cycler (Bio‐Rad, USA) to synthesize cDNA from the extracted RNA. qRT‐PCR was conducted on a QuantStudio 5 Real‐Time PCR System (Thermo Fisher Scientific, USA) with SYBR Green dye for fluorescent detection of amplification signals. The same RNA extraction and qRT‐PCR procedure were applied to RAW264.7 macrophages to assess the immunomodulatory effects induced by hydrogels with different compositions. The complete list of primer sequences is available in Table S1 (Supporting Information).

### Western Blot Analysis

Bone marrow–originated mesenchymal stem cells (BMSCs) were seeded at a density of 1 × 10^6^ cells per well in 6‐well plates and co‐cultured with titanium sheets coated with hydrogels containing different components for 7 days. Subsequently, total protein was extracted from the cells and subjected to Western blot analysis to evaluate the expression levels of osteogenesis‐related proteins (Col1, Runx2, ALP, BMP‐2). Similarly, HUVECs were seeded at the same density in 6‐well plates and co‐cultured with titanium sheets coated with hydrogels containing different components for 7 days. Total protein was then collected from the HUVECs for Western blotting was performed to evaluate the expression levels of angiogenesis‐related proteins (Hif‐1α, VEGF). MC3T3 cells were plated in six‐well culture plates at a density of 1 × 10⁶ cells per well. Following full attachment, the cells were treated with hydrogen peroxide (200 µm) for 30 min to induce oxidative stress. Subsequently, the cells were co‐cultured with titanium discs coated with different hydrogel formulations for 36 h. Total cellular proteins were then extracted and subjected to Western blot analysis to assess the expression levels of endogenous anti‐ROS‐related proteins(HO‐1, SOD1, SOD2).

Cell lysates were obtained using RIPA buffer (Beyotime, China), and the protein content was quantified with a BCA assay kit (Beyotime, Beijing, China) following the supplier's guidelines. Equivalent amounts of total protein were subjected to electrophoretic separation on 10% SDS–polyacrylamide gels and subsequently transferred to PVDF membranes. To minimize non‐specific adsorption, the membranes were incubated for 2 h at room temperature in Tris‐buffered saline containing 0.1% Tween‐20 (TBS‐T) and 5% (w/v) skim milk. Primary antibodies were applied and incubated overnight at 4 °C, followed by incubation with horseradish peroxidase (HRP)‐conjugated goat anti‐rabbit secondary antibodies for 1 h at 4 °C. Chemiluminescent signals were captured using an ECL substrate (Epizyme, China) according to the manufacturer's recommendations. Primary antibodies utilized in the experiments were supplied by Proteintech (Wuhan, China), and detailed information is provided in Table S2 (Supporting Information).

### In Vivo Experiments

All animal procedures were performed following the ethical guidelines approved by the Institutional Animal Care and Use Committee of Nantong University (Approval No. S20250210‐009). Ethical approval for all in vivo experiments was obtained in line with the ARRIVE standards and the UK Animals (Scientific Procedures) Act (1986), ensuring compliance with both institutional and national regulations concerning animal welfare.

To evaluate the degradation behavior, inflammatory response, and tissue residue of CMCSMA/GelMA‐based composite hydrogels in a subcutaneous implantation model, three formulations—C/GMA, C/GMA@SeNPs, and Lap‐C/GMA@SeNPs—were investigated. These hydrogels were prepared according to the procedures outlined in Methods. Rats were anesthetized using a portable multi‐channel anesthesia system, after which the dorsal region was shaved and disinfected under sterile conditions. A minor surgical incision was made dorsally to create a subcutaneous pocket, into which 100 µL of hydrogel was injected. In situ cross‐linking was immediately induced via ultraviolet irradiation to achieve photopolymerization. A gelatin‐based hydrogel served as the control. Each experimental group was evaluated using four Sprague‐Dawley (SD) rats, a total of 16 rats were used in the study. At pre‐established time points—2 and 4 weeks after implantation—the animals were humanely sacrificed, and the implanted hydrogels were carefully excised, freeze‐dried, and weighed to assess residual mass. Concurrently, surrounding tissues were harvested and fixed in 10% neutral buffered formalin at 4 °C for subsequent histological evaluation.

To evaluate the in vivo integration of implants with surrounding bone tissue, the study included four experimental groups designated as Ti, Ti‐C/GMA, Ti‐C/GMA@SeNPs, and Ti‐Lap‐C/GMA@SeNPs. Each group consisted of five SD rats, a total of 16 rats were used in the study. A standardized femoral defect model was established. Rats were administered anesthesia via a portable multi‐channel anesthetic delivery system, and the surgical area was shaved and disinfected prior to incision. The cylindrical defect measuring 1.0 mm in diameter was created at the distal femur using a surgical drill, with continuous irrigation of cold sterile saline to prevent thermal damage. The titanium implant was carefully inserted into the defect, and any additional experimental treatments were applied as required. The wound was then sutured immediately after implant placement. At specified time points post‐operation, following euthanasia, femoral specimens embedding the implants were collected and subjected to fixation in 10% neutral buffered formalin at 4 °C for later morphometric and histological examinations.

### Micro‐Computed Tomography (Micro‐CT) Analysis

Micro‐computed tomography of fixed femurs was conducted on the SkyScan 1174 V2 platform operating at 50 kV to generate high‐resolution 3D reconstructions. The micro‐CT scans were processed for volumetric reconstruction using NRecon (version 1.7.3.1) and subsequently interpreted in DataViewer (version 1.5.6.2). Reconstruction parameters were configured as support = 1, σ = 1.0, and implant threshold = 700, bone threshold = 205. Image features were extracted from the micro‐CT images according to the statistical distribution of grayscale intensity values for qualitative evaluation of bone regeneration in the vicinity of various titanium implants. Gray density values, which ranged from 0 to 100 as positive integers, were directly obtained from the micro‐CT image pixels. Additionally, in the micro‐CT reconstruction, a cylindrical region centered on the implant and extending 100 µm into the adjacent bone was selected as the region of interest (ROI). This ROI was located ≈3.0 mm below the epiphyseal line along the longitudinal axis and extending distally across 100 consecutive slices to quantitatively analyze newly formed bone tissue.

### Histological Analysis

Following fixation in formalin, decalcification was achieved by incubating the femoral samples in a 10% (w/v) EDTA solution adjusted to pH 7.4 for ≈30 days. The chelating solution was periodically renewed every three days to facilitate continuous ion exchange and promote complete demineralization. The specimens were then dehydrated through a graded ethanol series followed by xylene clearing and embedded in paraffin. Serial sections with a thickness of 5 µm were cut from the distal metaphyseal region of each femur. These sections were subjected to H&E, Masson's trichrome, and immunohistochemical (IHC) staining to assess tissue morphology and matrix composition. The bone tissues adjacent to the implant sites were histologically examined, and representative fluorescence micrographs were captured using an automated inverted microscope to characterize tissue morphology. Using Image‐Pro Plus (IPP) 6.0 software, within a 200 µm peri‐implant region, quantitative assessment was carried out to assess the spatial distribution of both newly formed and mature bone. The extent of bone‐to‐implant contact (BIC) was expressed as the proportion of the implant surface contiguous with mineralized bone matrix.

### Statistical Analyses

Quantitative datasets were statistically evaluated using SPSS software (v18.0; IBM, Chicago, USA), and all quantitative results are presented as mean ± standard deviation (SD). Differences among the experimental groups were determined through one‐way analysis of variance (ANOVA), with subsequent pairwise comparisons conducted using the LSD post hoc test. Significance was defined as ^*^ *p* < 0.05, ^**^ *p* < 0.01, and ^***^ *p* < 0.001, while non‐significant differences are denoted as ns.

## Conflict of Interest

The authors declare no conflict of interest.

## Supporting information



Supporting Information

## Data Availability

The data that support the findings of this study are available from the corresponding author upon reasonable request.
