# Supplementary material for: Immunomodulatory Hydrogel Coating with SeNPs and Lithium Silicate Synergistically Promotes Osseointegration and Prevents Infection on Titanium Implants
Source: Adv Sci (Weinh). 2025 Dec 12;13(12):e13195. doi: 10.1002/advs.202513195 (PMC12948283; doi:10.1002/advs.202513195)

Supporting Information

**Immunomodulatory Hydrogel Coating with SeNPs and Lithium Silicate Synergistically Promotes Osseointegration and Prevents Infection on Titanium Implants**

*Su Jiang 1,#, Baisheng Cai 1,#, Cong Ye 1, #, Kefan Wu1, Kuan Liu1, Pengcheng Xu 1, *, Fan Liu1, *, and Yake Liu1, *.*

*1 Department of Orthopaedics, Affiliated Hospital of Nantong University, Medical School of Nantong University, Nantong 226001, China.*

*2 Binhai County People's Hospital,* *Yancheng,* *Jiangsu,* *224500,* *China.*

*3 Yancheng Dafeng People's Hospital, Yancheng,* *Jiangsu,* *224100, China.*

*4 Department of Orthopaedics, The First Affiliated Hospital of Huzhou University, Huzhou 313000, China.*

*# These authors contributed equally to this work.*

**Corresponding author*

Email: yakerliu@ntu.edu.cn

**Keywords:** anti‐infective properties, bone regeneration, implant coating, lithium magnesium silicate, SeNPs

| Raw 264.7 | | |
| --- | --- | --- |
| Gene |  | Primer sequence |
| iL-1β | Forward | CTACCTGTGTCTTTCCCGTG |
|  | Reverse | TTTGTTGTTCATCTCGGAGC |
| Arg-1 | Forward | ATCAACACTCCCCTGACAACC |
|  | Reverse | TCGCAAGCCAATGTACACGAT |
| iNOS | Forward | ACGCTTCACTTCCAATGCAAC |
|  | Reverse | CAGCCTCATGGTAAACACGTTC |
| iL-10 | Forward | GAGAAGCATGGCCCAGAAATC |
|  | Reverse | GAGAAATCGATGACAGCGCC |
| CCR7 | Forward | AGAGGCTCAAGACCATGACGGA |
|  | Reverse | TCCAGGACTTGGCTTCGCTGTA |
| CD206 | Forward | CTCGTGGATCTCCGTGACAC |
|  | Reverse | GCAAATGGAGCCGTCTGTGC |
| GAPDH | Forward | AGAACATCATCCCTGCATCCAC |
|  | Reverse | TCAGATCCACGACGGACACA |
| hMscs | | |
| Gene |  | Primer sequence |
| ALP | Forward | CGTCTCCATGGTGGATTATGCT |
|  | Reverse | CCCAGGCACAGTGGTCAAG |
| Runx2 | Forward | TCTTCCCAAAGCCAGAGCG |
|  | Reverse | TGCCATTCGAGGTGGTCG |
| OPN | Forward | CCAAGCGTGGAAACACACAGCC |
|  | Reverse | GGCTTTGGAACTCGCCTGACTG |
| OCN | Forward | GCCCTGACTGCATTCTGCCTCT |
|  | Reverse | TCACCACCTTACTGCCCTCCTG |

**Table S1.** Primer sequences for qRT-PCR amplification of the target genes

Target Forward Reverse.

**Table S2.** The antibody used in WB and IF of this study.

|  | PRODUCT | Manufacturer | Catalog | Applications |
| --- | --- | --- | --- | --- |
| Primary antibody for WB | Col1 | Proteintech | No. 14659-1-AP | WB |
|  | ALP | Proteintech | No. 11187-1-AP | WB |
|  | Runx2 | Proteintech | No. 20700-1-AP | WB |
|  | BMP-2 | Proteintech | No. 66383-1-lg | WB |
|  | VEGF | Proteintech | No. 22341-1-lg | WB |
|  | iNOS | Proteintech | No. 22226-1-lg | WB |
|  | HO-1 | Proteintech | No.10701-1-AP | WB |
|  | SOD1 | Proteintech | No.10269-1-AP | WB |
|  | SOD2 | Proteintech | No.24127-1-AP | WB |
|  | Arg-1 | Proteintech | No. 16001-1-lg | WB |
|  | HIF-1α | Proteintech | No. 20960-1-AP | WB |
|  | Beta-Actin | Proteintech | No. 20536-1-AP | WB |
|  | GAPDH | Proteintech | No. 10494-1-AP | WB |
| Secondary Antibody for WB | Multi-rAb HRP-Goat Anti-Rabbit Recombinant Secondary Antibody (H+L) | Proteintech | No. RGAR001 | WB |
| Primary antibody for IF | OPN | Proteintech | No. 22952-1-AP | IF |
|  | Arginase-1(Arg-1) | Proteintech | No. 16001-1-AP | IF |
|  | iNOS | Proteintech | No. 80517-1-RR | IF |
| Secondary antibody for WB | CoraLite488-conjugated Goat Anti-Rabbit IgG(H+L) | Proteintech | No. SA00013-2 | IF |

**Figures:**


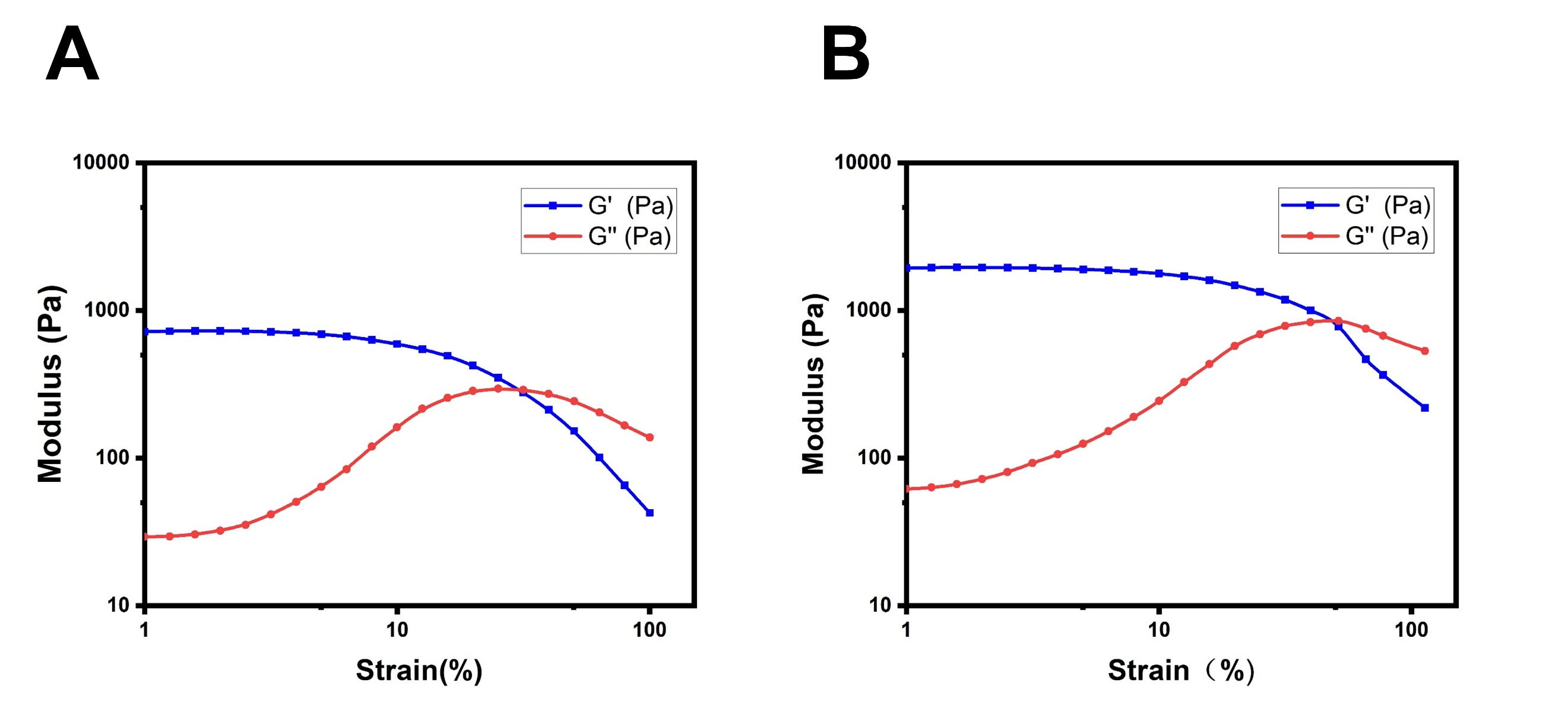


**Figure S1.** Characterization of hydrogel coatings. **(A,B)** Rheological behavior of C/GMA and C/GMA@SeNPs hydrogels.


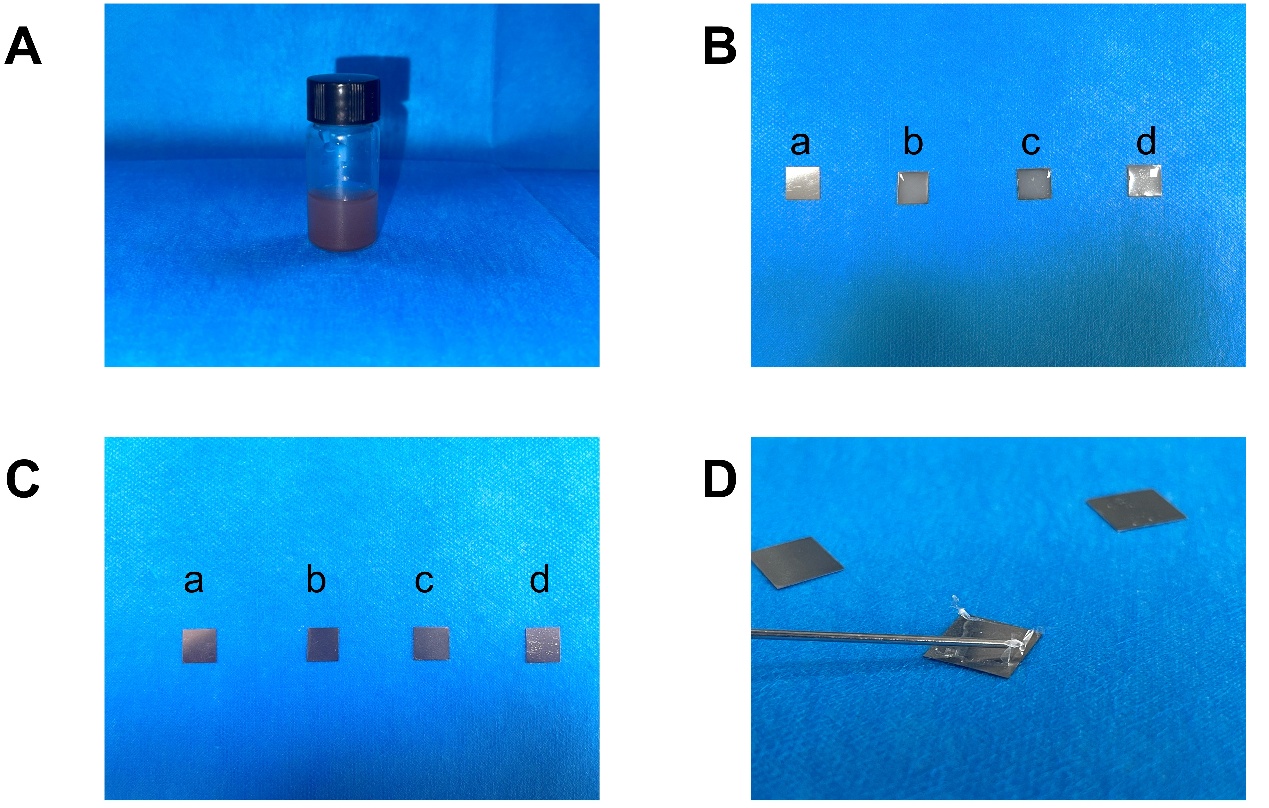


**Figure S2.** Materials and preparation process of hydrogel implant coatings. (A) Selenium Nanoparticles (SeNPs) Stock Solution. (B) Titanium plates coated with different hydrogel formulations: a (pure titanium sheet), b (Ti-C/GMA), c (Ti-C/GMA@SeNPs), and d (Ti-Lap-C/GMA@SeNPs). (C) Photo-cured titanium plates with respective hydrogel coatings, grouped as in panel B. (D) Hydrogel coatings exposed after aggressive wear testing.


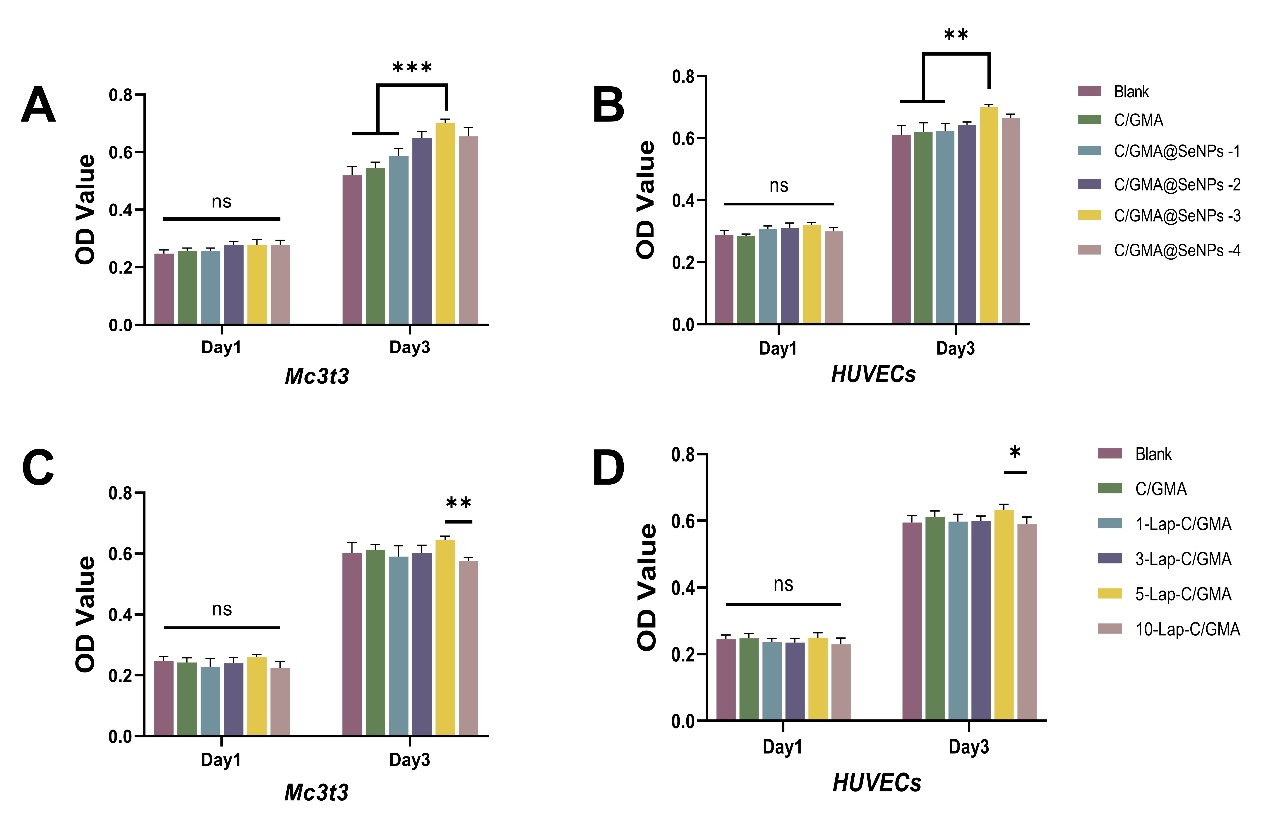


**Figure S3.** Cell viability was assessed using the CCK-8 assay following exposure to varying concentrations of the test agents. (A, B) illustrate the effects of different selenium nanoparticle (SeNP) concentrations on the viability of MC3T3 and HUVEC cells, respectively. (C,D) depict the corresponding effects of lithium magnesium silicate (Lap) at various concentrations on the same cell types. (n = 3; data are expressed as mean ± SD. *P < 0.05, **P < 0.01, ***P < 0.001, ns indicates no significant difference).


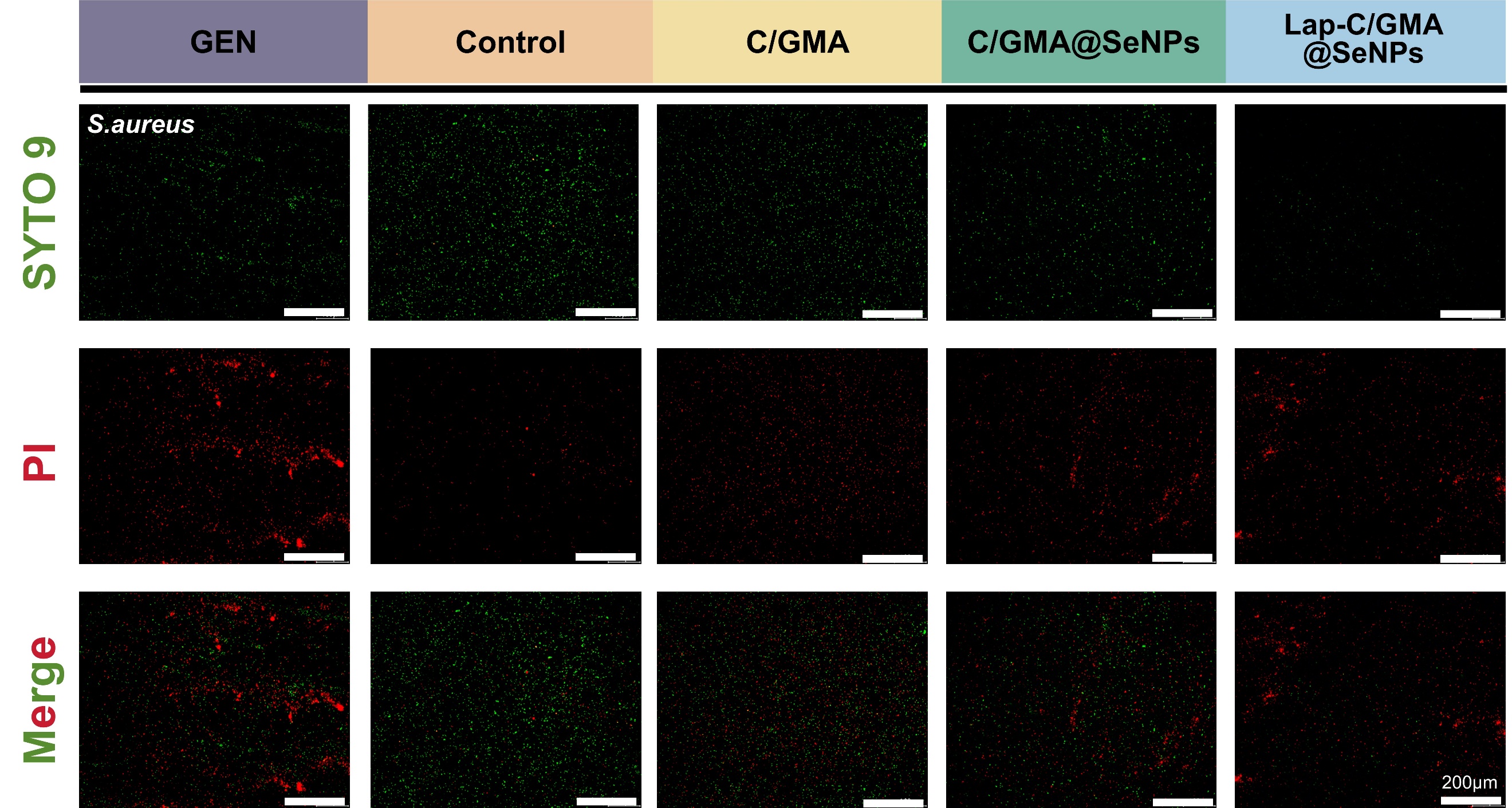


**Figure S4.** Live/dead staining images of *S. aureus* after co-incubation with different hydrogel coatings, including the blank control group and the gentamicin-treated group. (n = 3; data are expressed as mean ± SD. *P < 0.05, **P < 0.01, ***P < 0.001, ns indicates no significant difference).


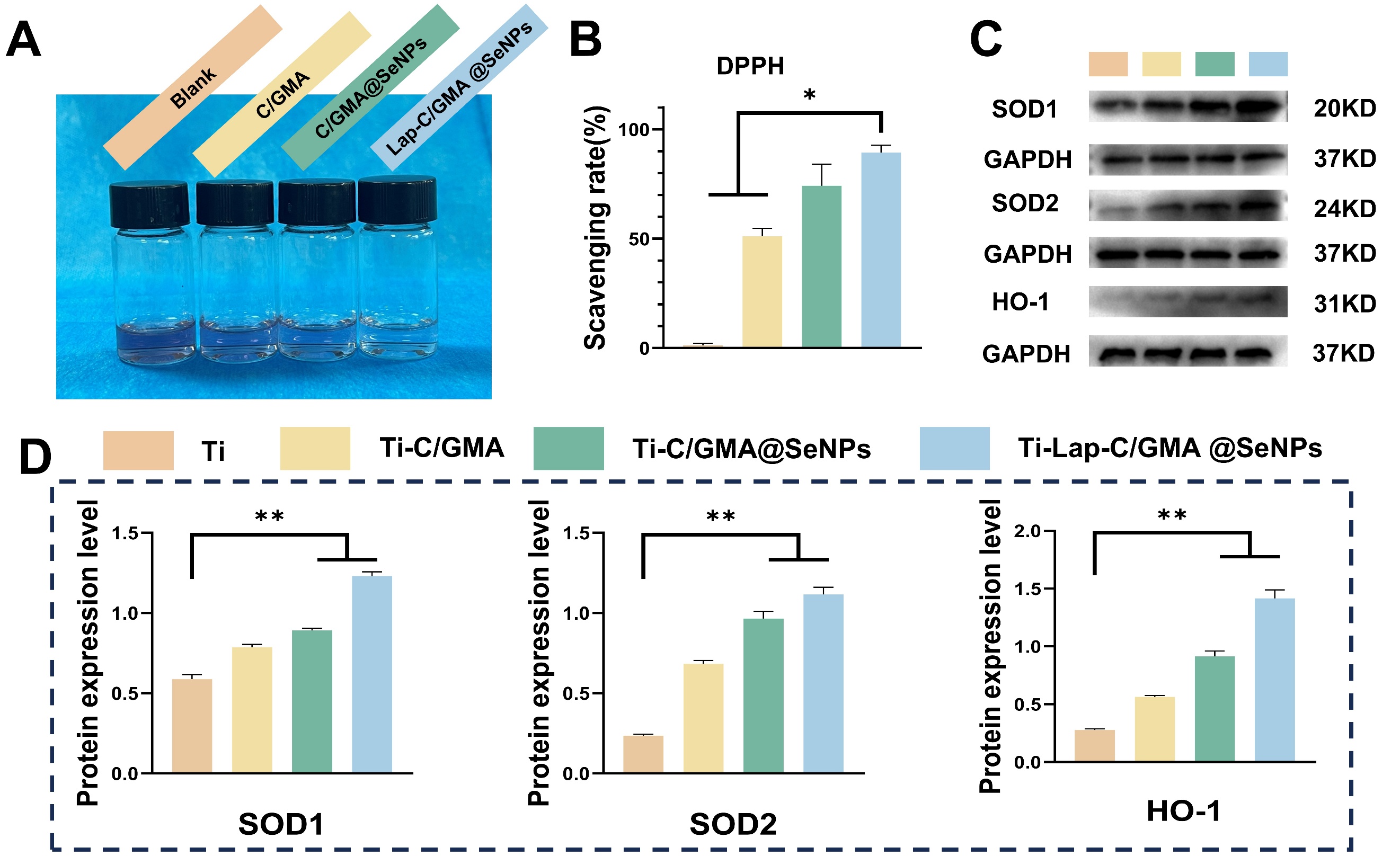


**Figure S5.** Evaluation of the antioxidative capacity of hydrogel coatings. (A, B) DPPH radical scavenging assay and corresponding quantitative analysis of different hydrogel compositions.(C, D) Western blot (WB) results and quantitative analysis of various hydrogel groups. (n = 3; data are expressed as mean ± SD. *P < 0.05, **P < 0.01, ***P < 0.001, ns indicates no significant difference).


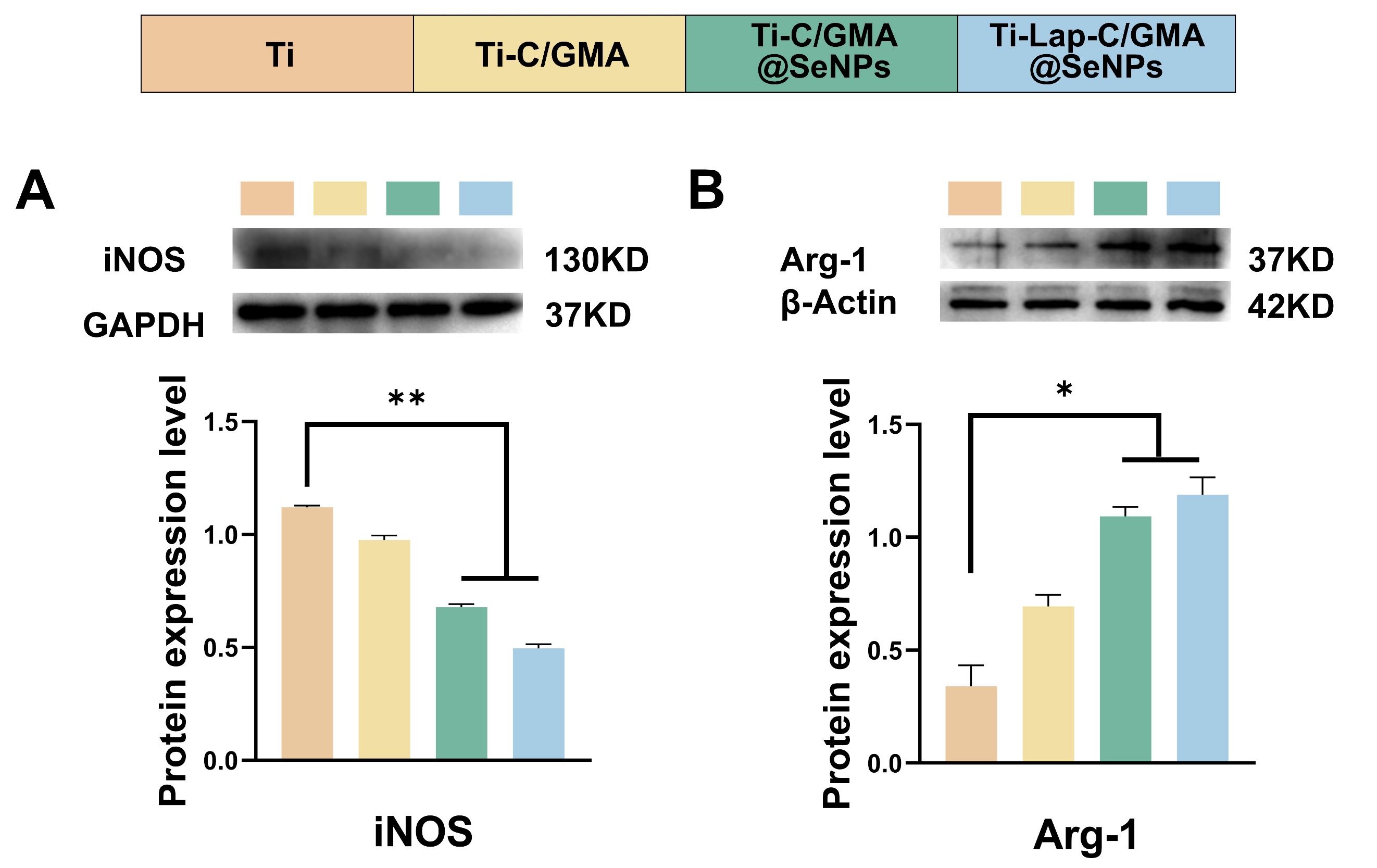


**Figure S6.** Western blot (WB) detection and quantitative analysis of iNOS and Arg-1 expression in RAW264.7 cells co-cultured with titanium sheets coated with hydrogels of varying compositions. (n = 3; data are expressed as mean ± SD. *P < 0.05, **P < 0.01, ***P < 0.001, ns indicates no significant difference).


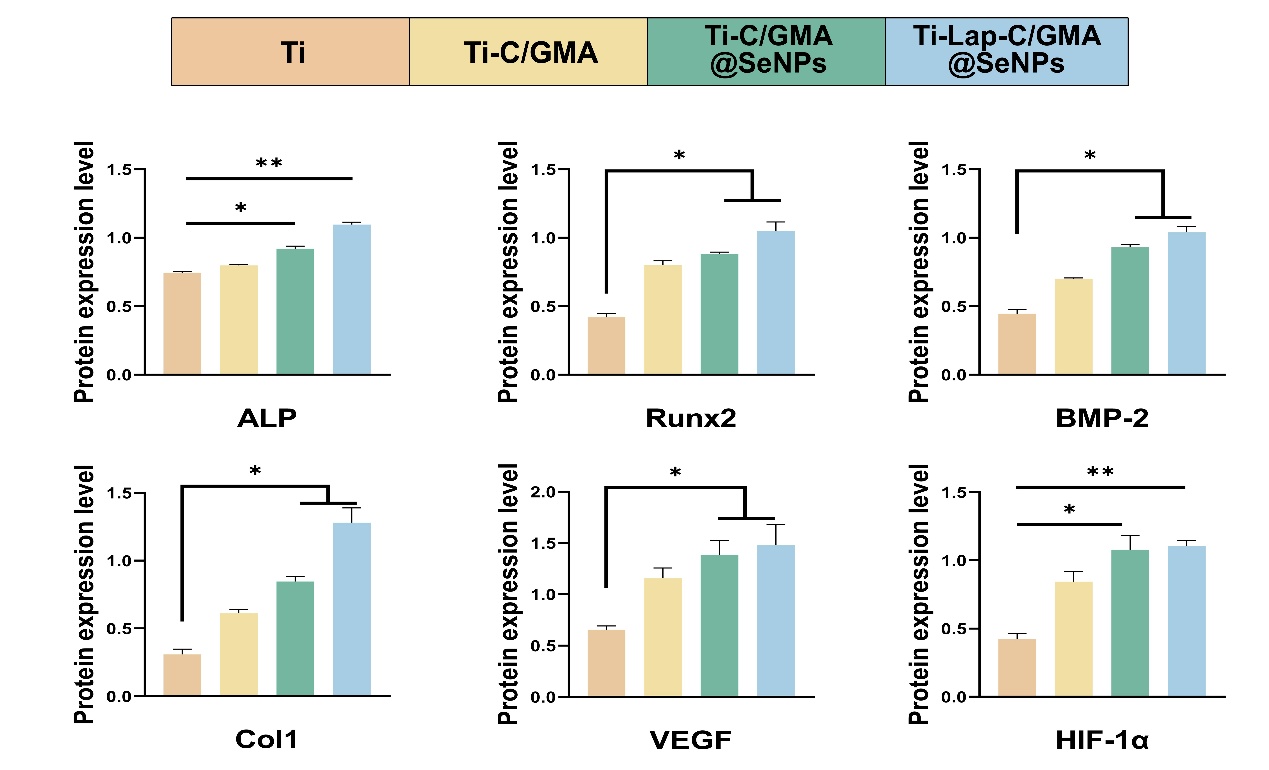


**Figure S7.** Western blot analysis was performed to evaluate the protein expression profiles of bone marrow-derived mesenchymal stem cells (BMSCs) and human umbilical vein endothelial cells (HUVECs) following 7-day co-culture with hydrogel-coated titanium plates. (n = 3; data are expressed as mean ± SD. *P < 0.05, **P < 0.01, ***P < 0.001, ns indicates no significant difference).


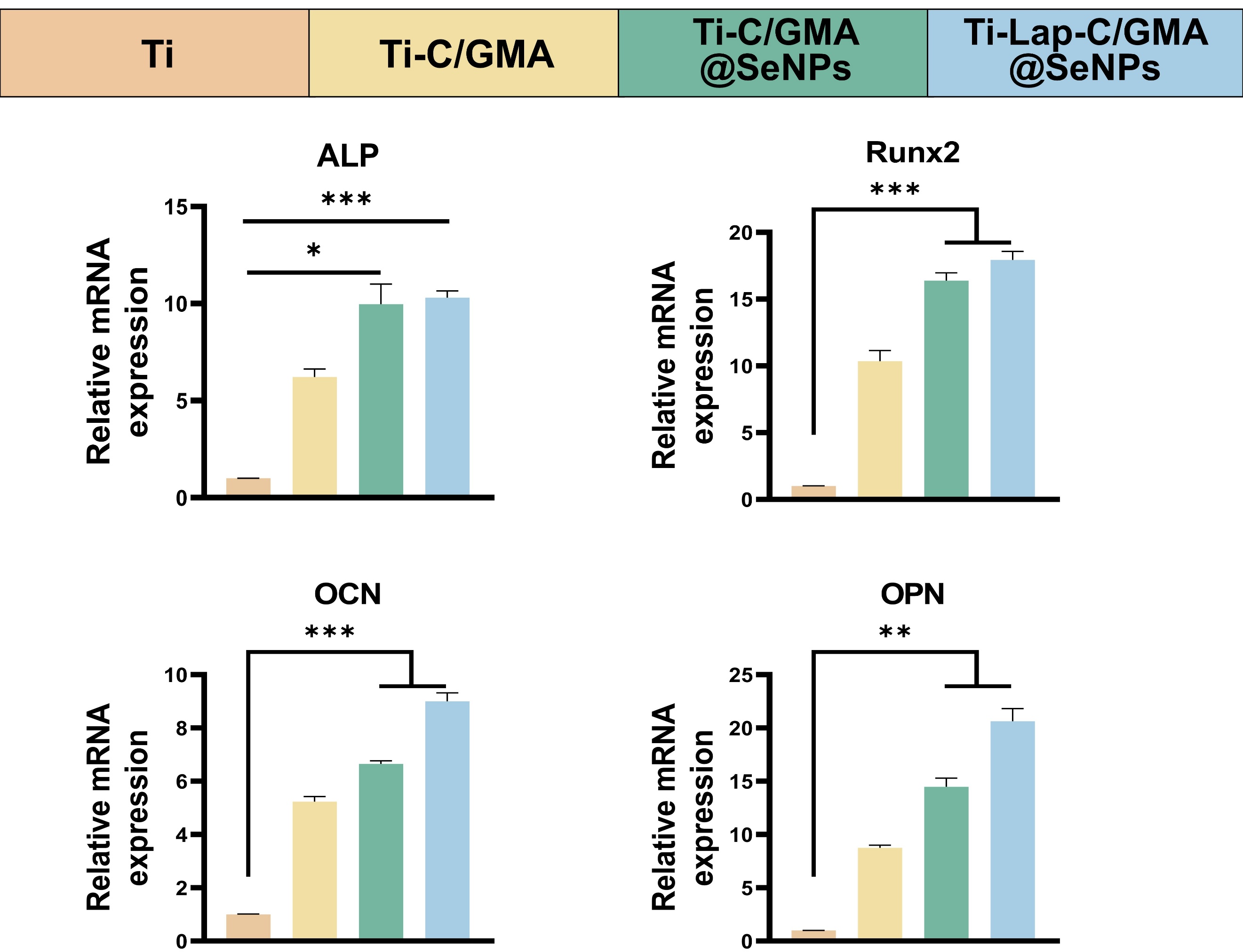


**Figure S8.** The expression levels of osteogenesis-related genes (ALP, Runx2, OCN, and OPN) in hMSCs following co-culture with titanium substrates coated with different hydrogels.(n = 3; data are expressed as mean ± SD. *P < 0.05, **P < 0.01, ***P < 0.001, ns indicates no significant difference).


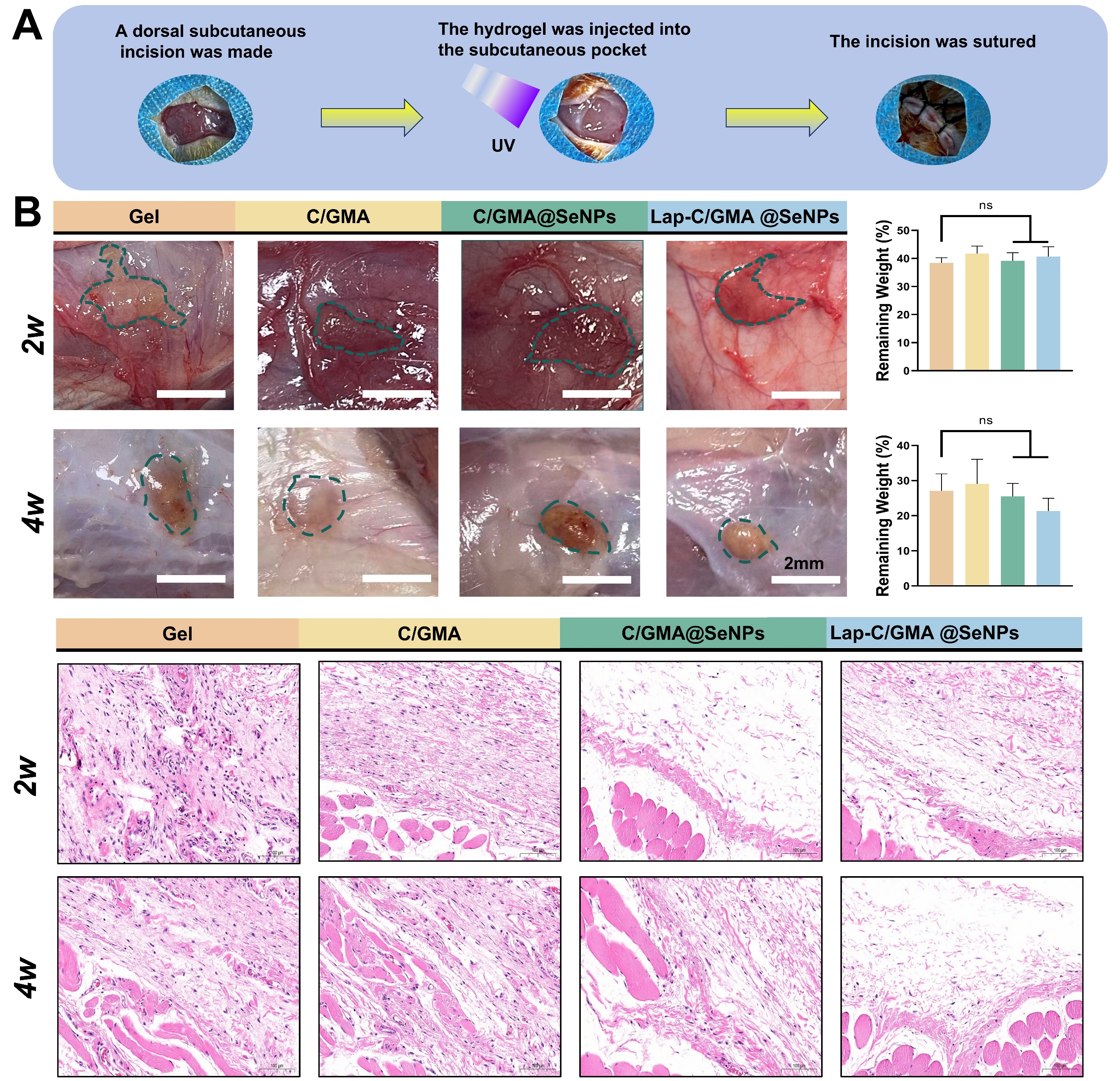


**Figure S9.** Subcutaneous implantation model to evaluate the degradation efficiency of hydrogels.(A) Schematic illustration of hydrogel implantation into subcutaneous pockets.(B) Representative macroscopic morphology, H&E staining images, and quantitative analysis of degradation rates of different hydrogel compositions after 2 and 4 weeks of subcutaneous implantation. (n = 4; data are expressed as mean ± SD. *P < 0.05, **P < 0.01, ***P < 0.001, ns indicates no significant difference).


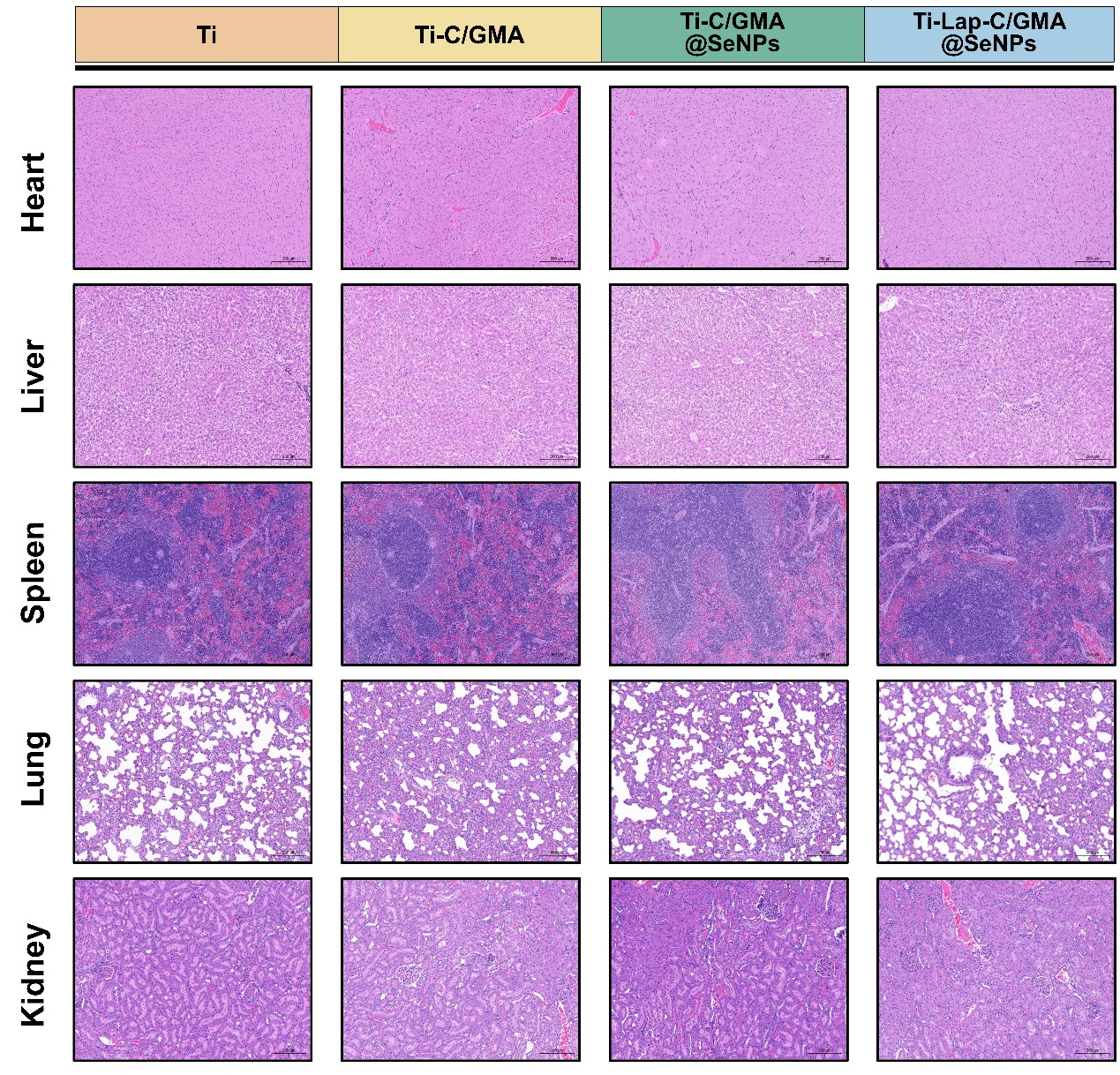


**Figure S10.** At 8 weeks post-implantation, heart, liver, spleen, lung, and kidney tissues from each group were collected and subjected to hematoxylin and eosin (HE) staining. (n=3).

**Raw Data of WB bands:**


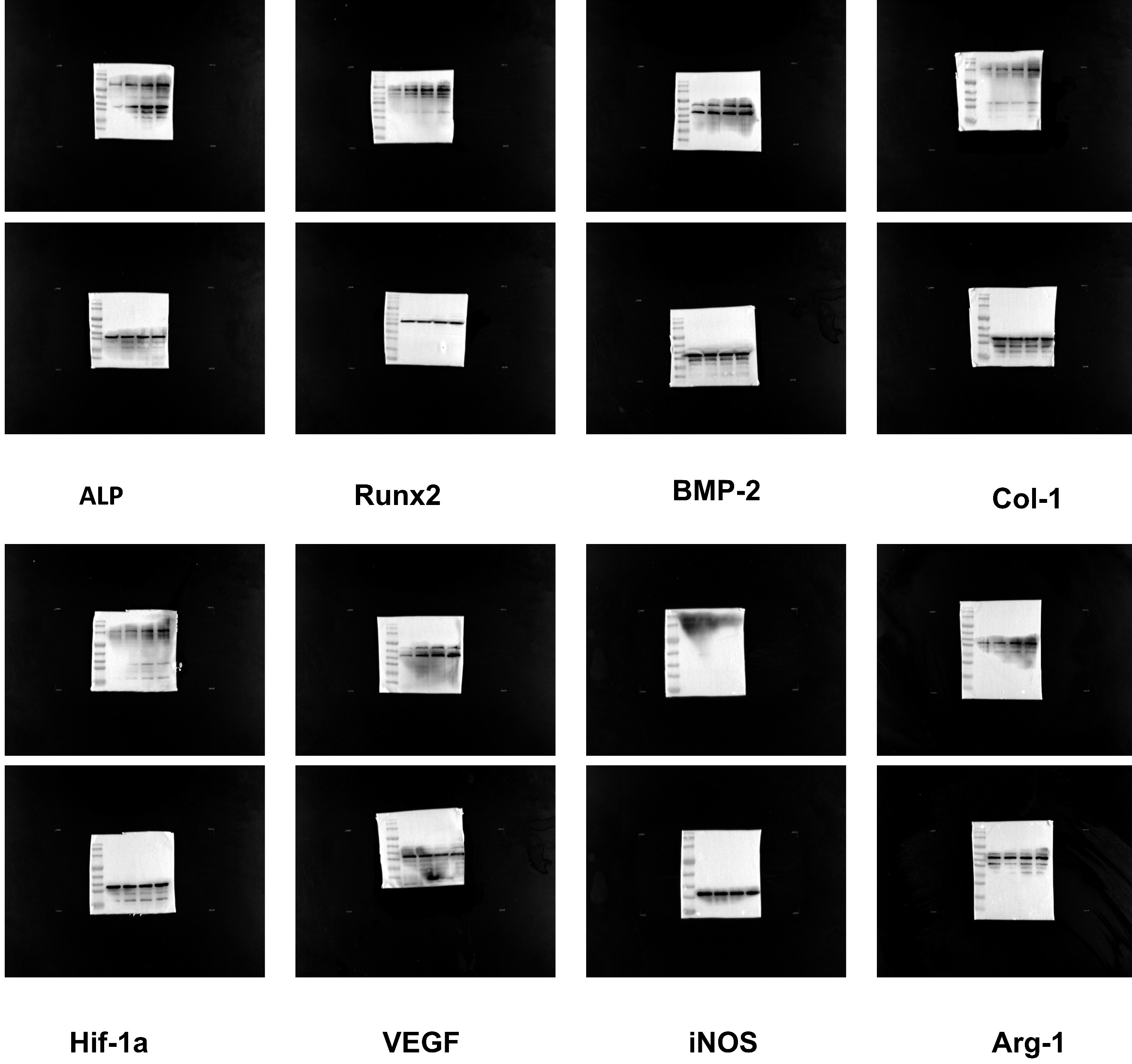


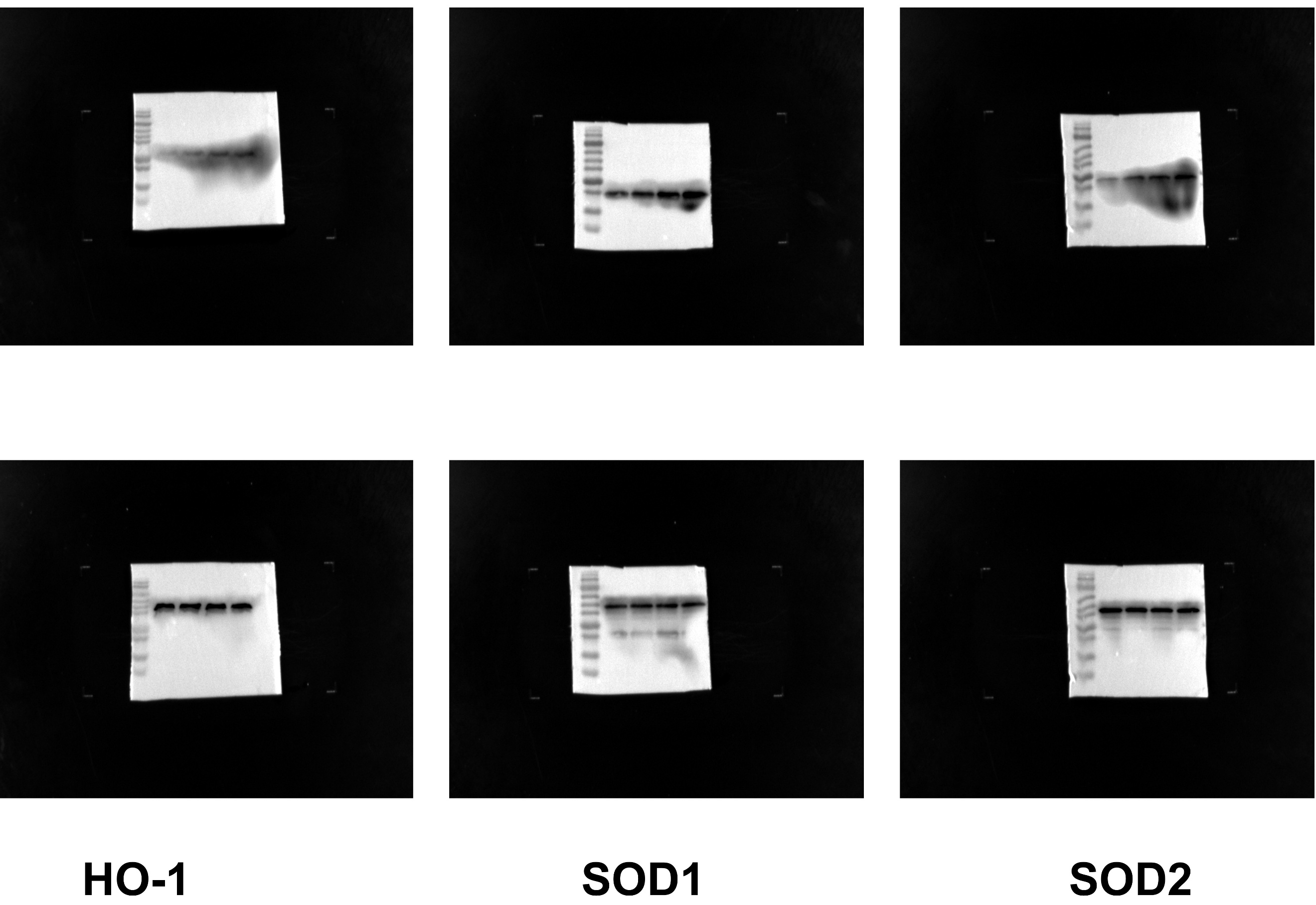

Supplement: Supplementary file 1 — Supporting Information [file ADVS-13-e13195-s001.docx]
